# An insight in proteome profiling of *Tuta absoluta* larvae after entomopathogenic fungal infection

**DOI:** 10.1038/s41597-022-01593-y

**Published:** 2022-08-19

**Authors:** Gurmeet Kour Bali, Sanjay Kumar Singh, Vinod Kumar Chauhan, Neha Joshi, Firdous Ahmad Bhat, Waseem Akram Malla, Boman Ramanujam, Richa Varshney, Manpreet Kour, Radhakrishnan Sitaram Pandit

**Affiliations:** 1 Department of Zoology, Savitribai Phule Pune University, Pune, 411007 India; 2 National Fungal Culture Collection of India, Biodiversity and Paleobiology Group, MACs Agharkar Research Institute, Pune, 411004 India; 3 Department of Zoology, Visva-Bharati University, Santiniketan, 731235 India; 4 Institute of Bioinformatics, International Technology Park, Bangalore, 560066 India; 5 Manipal Academy of Higher Education, MAHE, Manipal, 576104 India; 6 Division of Veterinary Biotechnology ICAR-Indian Veterinary Research Institute, Izatnagar, Bareilly, 243122 India; 7 ICAR- National Bureau of Agricultural Insect Resources, Bengaluru-560024, Karnataka India; 8 Division of Veterinary Medicine, Faculty of Veterinary science and Animal Husbandry, S.K. University of Agricultural Sciences and Technology, R.S Pura, Jammu 181102 India

**Keywords:** Immunology, Molecular biology

## Abstract

*Tuta absoluta* (L.) (Lepidoptera: Gelechiidae), a major pest of solanaceous plant species, causes serious losses in the agriculture sector around the globe. For better pest management, entomopathogenic fungi such as *Beauveria bassiana* and *Purpureocillium lilacinum*, play an efficient role in suppressing the pest population. The present study was carried out to analyse the effects post fungal infections through proteome profiling using an Orbitrap Fusion Tribrid mass spectrometer. A total of 2,201 proteins were identified from the fourth instar larvae of *T. absoluta*, of which 442 and 423 proteins were significantly dysregulated upon infection with *P. lilacinum* and *B. bassiana* respectively. The potential proteins related to immune systems as well as detoxification processes showed significant alterations after post fungal infection. Studies on *T. absoluta* proteomics and genomics as well as the consequences of entomopathogenic fungal infection on the immune response of this insect could provide an initial framework for exploring more fungus-host interactions for the development of better strategies for integrated pest management.

## Abstract (summary)

**Table:** 

Measurement(s)	Proteome
Technology Type(s)	Mass Spectrometry
Factor Type(s)	Dosage
Sample Characteristic - Organism	Tuta absoluta

## Background & Summary

*Tuta absoluta* (Meyrick) (Lepidoptera: Gelechiidae) is an oligophagous pest causing serious damage to solanaceous crop plants, especially tomato (*Solanum lycopersicum* L.)^1,2^. With the increase in global trading and lack of quarantine measures, *T. absoluta* has spread to almost every tomato producing country, thus threatening 87% of tomato production across the globe^3–5^. As this pest has a high fecundity rate with overlapping generations, which often leads to selection pressure, *T. absoluta* has developed resistance to numerous insecticides^6,7^. To overcome the problem of chemical insecticides and to eradicate pest infestation, entomopathogenic microbes (biological control) can play a significant role in reducing insect populations and avoiding damage from chemical residues, which have detrimental effects on humans and the environment^8^. Among entomopathogenic microbes, entomopathogenic fungi can be considered promising agents to control various insect pests. The generalist entomopathogenic fungi, *B. bassiana* and *P. lilacinum* cause pathogenicity to insect hosts by breaching their cuticle and degrading insect cuticular proteins, chitin, and lipids, which are hydrocarbons, in nature^9–14^. After invasion, the hyphae of the fungal pathogen perforate the haemocoel of the insect body and proliferate throughout the insect host to form hyphal bodies after replication^15,16^. During the process of invasion, entomopathogenic fungi secrete many secondary metabolites that are toxic and immunosuppressive and often lead to the decline of the host defence system, leading to the death of insects^17,18^. To combat the process of colonization by *B. bassiana*, the insect reciprocates by triggering the melanization process, producing antimicrobial peptides (AMPs), detoxifying enzymes and reactive oxygen species^19–21^. The evolutionary process has favored to the development of insects to develop a more potent and efficient immune system in insects to combat invading pathogens and parasites in their surroundings^22,23^. These pathogens trigger innate immune responses after invasion, which comprise cellular and humoral immune reactions.

The present study was carried out to identify proteins in *T. absoluta* and their alterations upon entomopathogenic fungal infection with an advanced and robust proteomic technique using an Orbitrap Fusion Tribrid mass spectrometer. A comprehensive analysis was carried out to elucidate the potential proteins involved in the host immune response and detoxification by the *T. absoluta* larvae. These potential proteins have shown significant alterations after entomopathogenic fungal infection. The list of potential proteins is provided in Supplementary Tables 1 & 2 and were obtained after 48 h of infection with *B. bassiana* and *P. lilacinum*.

**Fig. 1:**
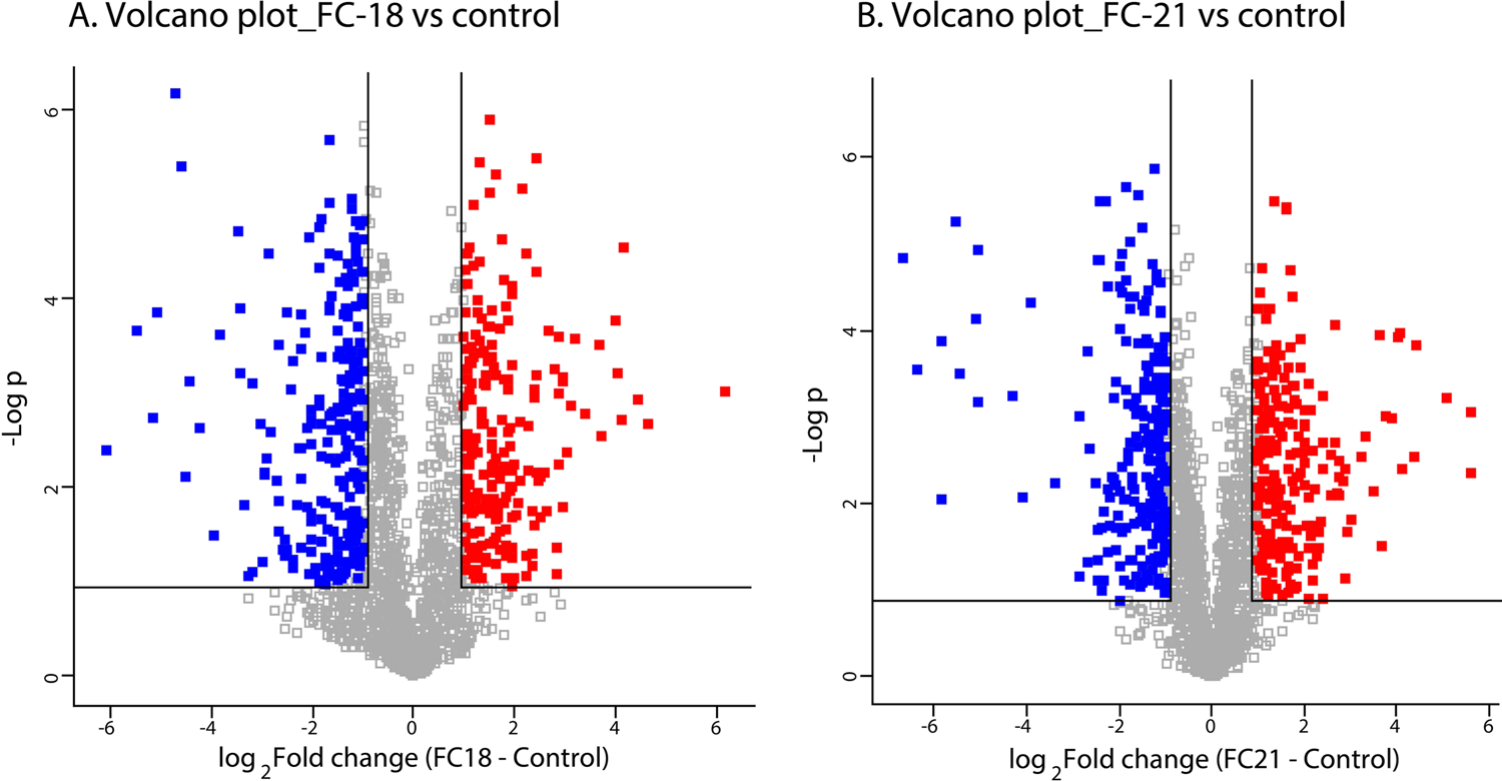
Volcano plots showing the distribution of proteins in (**A**) *Tuta absoluta* infected with *P. lilacinum* (FC18) when compared with the control sample. (**B**) *Tuta absoluta* Infected with *B. bassiana* (FC21) when compared with control sample, dysregulated proteins were highlighted in red (overexpressed) and blue (downregulated).

**Fig. 2:**
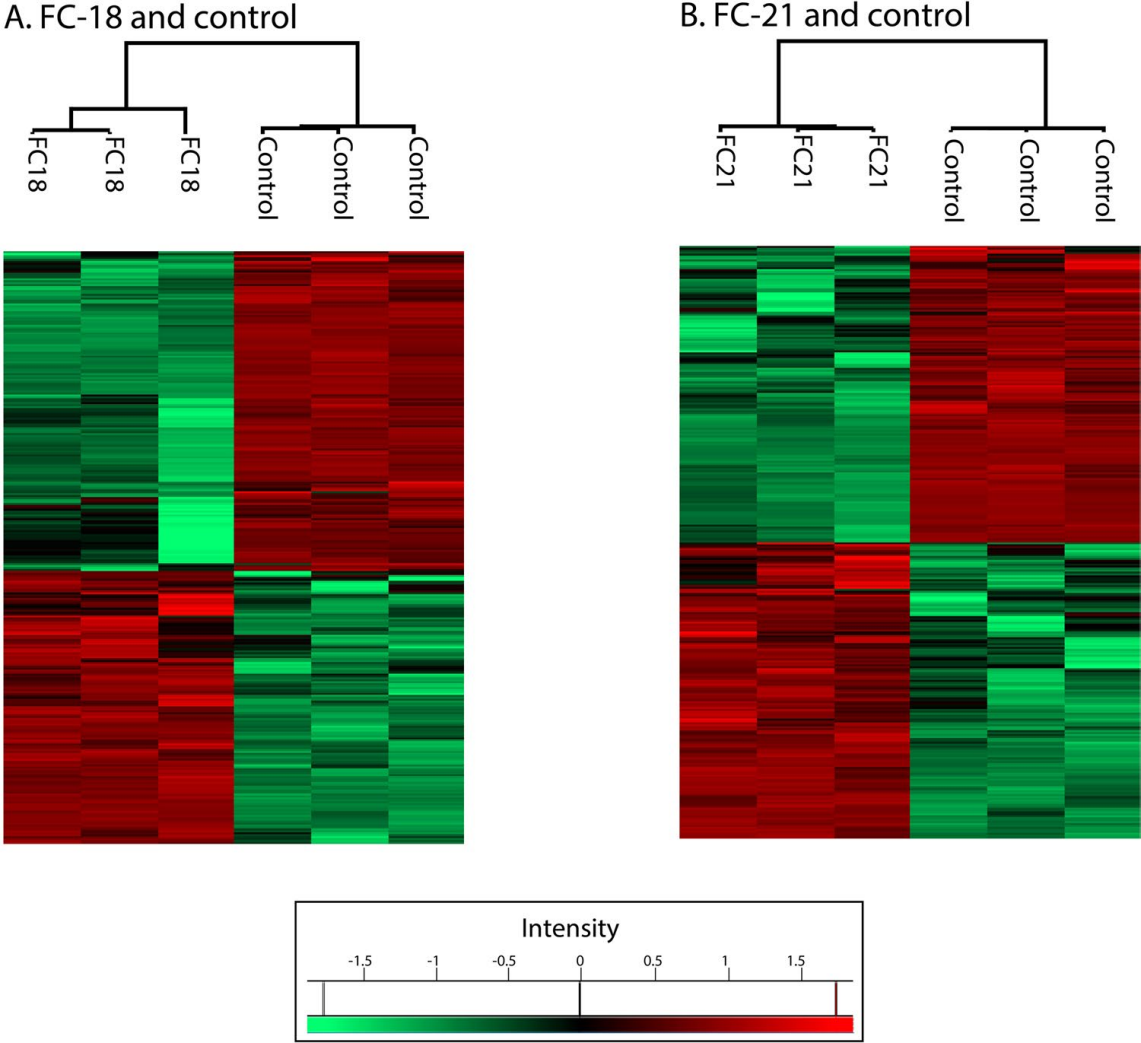
Clustering of treated samples and control sample separately in heatmap (**A**) *P. lilacinum* (FC18) infected larvae and control. (**B**) *B. bassiana* (FC21) infected larvae and control. Red colors indicate the intensity of expression of the protein in the sample and green color represents low expression level.

The raw data were obtained from mass spectrometry analysis against the protein databases of *Manduca sexta* and *Plutella xylostella*, and a total of 2,201 proteins were identified in *T. absoluta*. Among these, 1,440 proteins mapped to *M. sexta*, whereas 878 proteins were mapped from the *P. xylostella* protein database (http://www.insect-genome.com/data/detail.php?id=19) (Supplementary data file 1). Dynamic changes in the expression of proteins indicate that *B. bassiana* and *P. lilacinum* were potent and altered the protein expression in fourth instar larvae of *T. absoluta* 48 h post-infection. The pathogenicity of the entomopathogenic fungus *B. bassiana* resulted in the dysregulation (upregulation and downregulation) of 452 proteins, while *P. lilacinum* revealed 455 proteins in fourth instar larvae of *T. absoluta*. Upon infection with the entomopathogenic fungi, *P. lilacinum* and *B. bassiana*, there was a significant dysregulation at the protein expression level in *T. absoluta*. The distribution of proteins is shown by volcano plots, where upregulated and downregulated proteins are highlighted in red and blue, respectively (Fig. 1A,B; *P. lilacinum*-FC 18 and *B. bassiana-*FC21). Using a threshold of a 2-fold change (FC) difference and <0.05 FDR (adjusted p value), 442 proteins were identified as dysregulated in *P. lilacinum*-infected larvae when compared with control samples (Supplementary data file 2). Similarly, *B. bassiana*-infected samples revealed 423 significantly dysregulated proteins in comparison to control samples (FC > 2, FDR < 0.05 p) (supplementary data file 3). Heatmaps for significantly dysregulated proteins show clustering of the two types of treated samples separately from the control samples (Fig. 2A,B).

Gene ontology enrichment analysis showed that both entomopathogenic fungi affected the biological, cellular and molecular processes in *T. absoluta*. These differentially expressed proteins were involved in various metabolic activities, including oxidation, hydrolysis and conjugation, catalytic activity, etc., which are activated by enzymes with the resultant release of respective metabolites^24,25^. The bioinformatic analysis revealed many proteins and their interactions with other associated members. The data obtained will provide significant insights into understanding the mechanism of the pathogenicity of *B. bassiana* and *P. lilacinum* and their interaction with *T. absoluta* larvae.

## Methods

### Insect culture rearing

Adults of *T. absoluta* were procured from the Germplasm Division, ICAR-NBAIR-Bangalore, India and released into a large sterile breeding chamber with tomato seedlings. The insect culture was maintained under laboratory conditions at 27 ± 2 °C and 55% R.H. The moths were supplied with 10% honey solution as a source of food. Eggs were collected from the breading chamber and reared in rearing jars containing tomato leaves for growth and development. The early 4th instar larvae were collected from rearing jars for treatment with the entomopathogenic fungi *B. bassiana* and *P. lilacinum*.

### Entomopathogenic fungal strain collection and identification

*B. bassiana* and *P. lilacinum* were isolated from soil samples from the agricultural fields of Pune University, Pune, India and were identified on the basis of morphology and sequencing with phylogenetic analysis. Sequences have been submitted to the National Center for Biotechnology Information (NCBI) GenBank with accession numbers MT672601 (*Purpureocillium lilacinum*) and MT672634. (*Beauveria bassiana*).

### Preparation of fungal conidial suspension for infection

*In vitro* cultures of *B. bassiana* and *P. lilacinum* were raised following a standard procedure on potato dextrose agar plates. The inoculated plates were incubated and maintained at a temperature of 25 ± 1 °C with 70 ± 5% relative humidity for 2 weeks. The spores and hyphae were scraped from a fully grown colony and transferred to sterile water containing 0.01% (vol/vol) Tween-20. The conidia were dispersed by vortexing, and the hyphae were removed by filtering the suspension with a sterilized muslin cloth. The concentration of conidia was measured using a haemocytometer. A final concentration of 1 × 10^7^ conidia mL^−1^ with sterile 0.01% Tween-20 solution was used for the experiments.

### Infection of *T. absoluta* larvae with *B. bassiana* and *P. lilacinum* strains

The fourth instar larvae of *T. absoluta* were infected with *B. bassiana* and the *P. lilacinum* strain separately at a concentration of 1 × 10^7^ conidia mL^−1^ by the dipping method for 15 sec. For each treatment, 100 larvae were immersed in the conidial suspension and then released onto tomato leaves after removing extra spores. The larvae in the control set were treated with sterile distilled water containing 0.01% Tween-20.

### Sample preparation for proteomic analysis

Whole fourth instar larvae were collected from both infected and control sets 48 h post infection. One hundred larvae from each group were ground to a fine powder in liquid nitrogen using a prechilled mortar and pestle. To this, 500 µl extraction buffer (0.5 M Tris-HCl (pH 7.5); 50 mM EDTA; 0.5 M sucrose; 0.1 M KCL and 2% *β*-mercaptoethanol) was added and vortexed for 10 min. The homogenate was centrifuged at 10,000 rpm for 10 min, and the supernatant was transferred to a new tube. To the supernatant, an equal amount of water-saturated phenol was added, and the mixture was vortexed well for 10 min, and centrifuged at 10,000 rpm for 10 min. The phenol phase was retracted and precipitated with 5 volumes of 0.1 M ammonium acetate. After 24 h, the precipitate was centrifuged at 10,000 rpm for 10 min, and the supernatant was discarded. To the pellet, 200 µl of 0.1 M ammonium acetate was added, the mixture was vortexed and again centrifuged at 10,000 rpm for 5 mins. This process was repeated twice. Then, the obtained pellets were washed with 200 µl of 100% acetone, air dried, suspended in 8 M urea and stored at −70 °C for further use. The entire process of centrifugation and precipitation was carried out at 4 °C. The total proteins from three biological replicates were pooled and dried in a speed vac concentrator and stored at −80 °C until till further use.

### Preparation of samples for mass spectrometry

Dried samples were reconstituted in 50 mM triethylammonium bicarbonate buffer and processed for in solution digestion. Briefly, the protein concentration of the samples was measured by a Pierce^TM^ BCA Protein Assay Kit. Based on the protein concentration, equal protein amounts (~100 µg) from each sample were taken. Reduction of the samples was carried out using a final concentration of 5 mM dithiothreitol (DTT) at 60 °C for 30 mins followed by alkylation by 10 mM iodoacetamide (IAA) for 30 min in the dark at room temperature. Samples were digested using Promega sequencing grade modified Trypsin at a ratio of 1:20 (enzyme: protein) at 37 °C overnight. Peptides were then acidified by 1% formic acid (FA) and were cleaned by a Sep-Pak C_18_ cartridge. Eluted samples were dried in a Speed Vac and stored at −80 °C until further analysis.

### LC–MS/MS analysis

Data acquisition for digested samples was carried out on a Thermo Scientific^TM^ Orbitrap Fusion Tribrid mass spectrometer (Thermo Fisher Scientific, Bremen, Germany) interfaced with a Thermo Scientific EASY-nLC 1000 (Thermo Fisher Scientific, Germany). Peptides were reconstituted in 0.1% formic acid and loaded onto a trap column (Thermo Scientific^TM^ Acclaim^TM^ PepMap^TM^ 100 C18 LC Column, 75 µm × 2 cm, 3 µm). Peptides were resolved on an analytical column (Thermo Scientific^TM^ Acclaim^TM^ PepMap^TM^ 100 C_18_ LC Column, 75 µm × 50 cm, 2 µm) at a flow rate of 300 nl/min using an optimized linear gradient of 8–32% solvent B (0.1% formic acid in 100% ACN) over 100 min. The total run time for each replicate, including sample loading and column reconditioning, was 120 min. One microgram of peptide was used in each mass spectrometer run. The MS data acquisition was carried out from the 350–1600 m/z range using an Orbitrap mass analyser. The AGC target was set to 400,000 with an ion injection time of 50 ms and dynamic exclusion of 30 sec. Precursor ions were fragmented using high energy collision dissociation (HCD) (NCE 32%) and analysed using an Orbitrap mass analyser with a resolution of 15000. For MS/MS scans, the AGC target was set to 100,000 with an ion injection time of 100 ms. Samples were analysed in technical triplicates.

## Data Records

The mass spectrometry proteomics data have been deposited in the ProteomeXchange Consortium (http://proteomecentral.proteomexchange.org) using the PRIDE partner repository with the dataset identifier PXD029374^26–28^.

## Technical Validation

### Data analysis

The MS/MS raw data searches were carried out using SEQUEST search algorithms using Proteome Discoverer 2.2 (Thermo Fisher Scientific, Bremen, Germany) against RefSeq protein databases of *Manduca sexta* and *Plutella xylostella*. The search parameters involved carbamidomethylation at cysteine residues (+57.021 Da) as a fixed modification and oxidation of methionine (+15.995 Da) as a dynamic modification. The precursor and fragment mass tolerances were set to 10 ppm and 0.05 Da, respectively. Trypsin was specified as protease, and a maximum of two missed cleavages were allowed. An FDR of 1% was applied as a cut-off value for reporting identified peptides. All 9 raw files pertaining to the three samples were searched against databases. In the peptide and protein quantifier node of the proteome discoverer, we specified the total peptide intensity of each sample for normalization. This node calculates the sum of abundance values of all peptides identified in each raw file referred to as the channel hereafter. The channel with the highest total abundance is then used as a reference to normalize all other channels by a constant factor per channel. In the end, the total abundance was the same for all channels. The software groups the abundance values of each peptide across three technical replicates. For statistical analysis we used Perseus proteomics software (version 1.6.2.2), where Student’s T test was performed to calculate p values. A fold change cut-off of 1.5 was applied to determine significantly dysregulated proteins.

### Gene ontology analysis

**Fig. 3:**
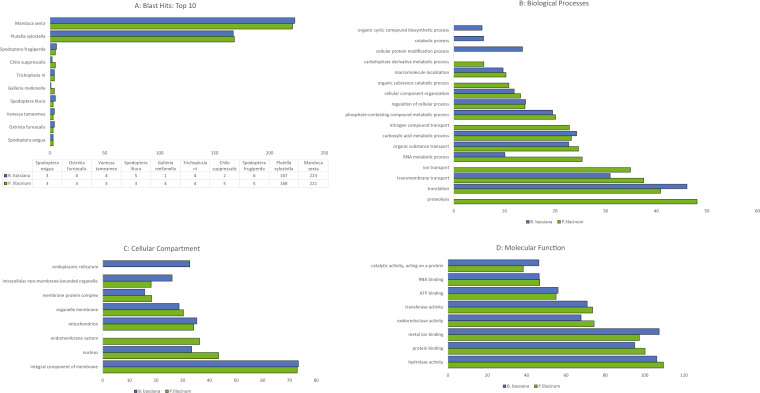
Gene ontology of dysregulated genes upon EPF infection of *P. lilacinum* and *B. bassiana* Bar chart showing the summary of Top 10 blast hits infected with *P. lilacinum* and *B. bassiana* datasets (**A**). Bar charts depicting the top enriched terms for Biological Processes (**B**), Cellular Compartment (**C**), and Molecular Functions (**D**). The detailed results are shown in Table 1.

Gene ontology analysis of differentially expressed proteins was performed by the Blast2GO^®^ software package (BioBam Bioinformatics Solutions, Spain). The protein sequences were imported into Blast2GO^®^ in Fasta format and aligned against the nonredundant (nr) NCBI database using the inbuilt Blast function (blastp) with an e-value cut-off of x10^−3^. The resulting hits were matched against the Gene Ontology Annotation database, and Gene Ontology was assigned to each hit on the basis of protein sequences using the mapping and annotation functions. The blast hits obtained were also subjected to Gene Ontology assignment based on protein domains and families using the InterProScan. The Gene Ontology results from the two steps were then merged together, followed by the generation of relevant graphs. The summary statistics of the annotation are provided in Table 1. The full list includes enriched GO terms for biological processes (BP), cellular components (CC) and molecular functions (MF) in *T. absoluta* infected with *P. lilacinum* and *B. bassiana* using blast2GO analysis along with the genes for each term. The topmost enriched GO terms for *P. lilacinum* and *B. bassiana* are summarized in Fig. 3 (A: Biological Processes; B: Cellular Compartments; C: Molecular Functions). Of the top enriched biological process terms, proteolysis, ion transport, nitrogen compound transport, organic substance catabolic process, and carbohydrate derivative metabolic process were enriched by when infection with *P. lilacinum*, whereas cellular protein modification process, catabolic process, and organic cyclic compound biosynthetic process were enriched after *B. bassiana* infection. From the uppermost enriched cellular component terms, the endomembrane system was enriched with *P. lilacinum* treatment, whereas in *B. bassiana* most of the proteins were localized to the endoplasmic reticulum. The top enriched molecular function was common to both datasets, although the enrichment score varied.

**Table 1:** GO annotations before and after merging in blast2GO in *P.lilacinum and B.bassiana*.

Summary of Merged Annotations	*P. lilacinum*	*B. bassiana*
Type		
GOs Before Merge	1351	1343
GOs After	1469	1442
Confirmed IPS GOs	2456	2658
Too General IPS GOs	251	263

GO annotations obtained before and after merging GO terms from core annotation and InterProScan annotation.

Furthermore, with the protein sequences and abundance values obtained from the mass spectrometry analysis, we used the abundance ratio column for the classification of upregulated and downregulated genes. The genes whose abundance ratio was greater than 1 with respect to the control were classified as upregulated genes, and those whose abundance ratio was less than 1 were considered downregulated genes. In addition, gene enrichment analysis of biological processes (upregulated and downregulated) of *T. absoluta* proteins was also performed separately after entomopathogenic fungal infection. *B. bassiana* (FC21) showed 213 upregulated genes and 210 downregulated genes and the genes involved in organic substance transport, protein localization to organelles etc., were upregulated, whereas those involved in cytoskeleton organization, actin filament-based processes etc., were downregulated (Supplementary Fig. 1i & ii). Similarly, *P. lilacinum* (FC18) infection resulted in 203 upregulated genes and 242 downregulated genes. Genes involved in organic substance transport, nucleotide-containing small molecule metabolic processes etc., were upregulated whereas cytoskeleton organisation, actin filament-based process etc. are downregulated (Supplementary Fig. 1iii & iv). Most importantly, through KEGG pathway analysis, we found that some of the proteins were involved in melanin biosynthesis (Supplementary Fig. 2). Melanin is known as an important defence molecule in invertebrate immunity^29,30^.

## Supplementary information

Supplementrary Information

## Data Availability

Perseus proteomics software version 1.6.2.2 (https://www.perseus-framework.org) was used for statistical analysis, where p values were calculated by Students t test. The volcano plots and heatmaps were also generated from the Perseus proteomics tools. The differentially expressed proteins were further subjected to Gene Ontology analysis using the Blast2GO^®^ software package (BioBam Bioinformatics Solutions, Spain) (https://www.biobam.com/omicsbox). These annotated genes were used for enrichment analysis of the gene ontologies of upregulated and downregulated genes by clusterProfiler version 4.2.2 (https://bioconductor.org).
