# Supplementary material for: An insight in proteome profiling of Tuta absoluta larvae after entomopathogenic fungal infection
Source: Sci Data. 2022 Aug 19;9:507. doi: 10.1038/s41597-022-01593-y (PMC9391459; doi:10.1038/s41597-022-01593-y)
Supplement: Supplementary file 1 — Supplementrary Information [file 41597_2022_1593_MOESM1_ESM.pdf]

Supplementary data Index

Supplementary data file 1. Screening of total proteins data obtained through LC-MS/MS which was analyzed by protein data base of *M. Sexta* and 878 from *P. xylostella* protein data base.

Supplementary data file 2. 2-fold change (FC) difference and <0.05 FDR adjusted p-value significance; the 455 proteins were identified as dysregulated from *P. lilacinum* infected larvae when compared with the control sample.

Supplementary data file 3. 2-fold change (FC) difference and <0.05 FDR adjusted p-value significance; the 452 proteins were identified as dysregulated from *B. bassiana* infected larvae when compared with the control sample.

Supplementary figure 1: Gene ontology enrichment analysis of biological process (upregulated and downregulated) of *T. absoluta* proteins after entomopathogenic fungal infection. Figure shows up regulated biological process (i) and down regulated biological process (ii) after *B. bassiana*-FC21 treatment; up regulated biological process (iii) and down regulated biological process (iv) after *P. lilacinum*-FC18 treatment. In figure A. Dot plot represents the up and regulated biological process in *T. absoluta;*.B. Heatmap represents the proteins involved in the up and down regulated biological process; C. Enrichment map represents the enriched biological process of up and down regulated proteins; D. Upset plot represents the up and down regulated biological process and the number of proteins common among the biological process.

Supplementary figure 2: KEGG pathway analysis proteins involved in amino acid tyrosine metabolism.

Supplementary table 1: Proteins identified which were involved in immune system of *T. absoluta* after *B. bassiana* infection and their expression profile after 48 h of post- infection.

Supplementary table 2: Proteins identified which were involved in immune system of *T. absoluta* after *P. lilacinum* infection and their expression profile after 48 h of post- infection.

Supplementary data file 1. Screening of total proteins data obtained through LC-MS/MS which was

analyzed by protein data base of *M. Sexta* and 878 from *P. xylostella* protein data base.

Accession no.	Protein	Organism	# Pept		# Unique Peptides	Fold-change difference
768443294	reticulon-1-A isoform X1 [Plutella xylostella]	Plutella	2	12	1	71.80
1714791788	LOW QUALITY PROTEIN: ryanodine receptor [Manduca sexta]	Manduca	11	26	5	25.03
1714785471	AMP deaminase 2 isoform X1 [Manduca sexta]	Manduca	6	7	1	17.47
768431272	calcium uniporter protein, mitochondrial like isoform X1 [Plutella xylostella]	Manduca and Plutella	1	10	1	16.47
1714843099	neurochondrin homolog [Manduca sexta]	Manduca	5	17	1	13.27
1194641147	phosphate carrier 1 [Plutella xylostella]	Plutella	6	62	1	10.69
768417308	sodium/potassium-transporting ATPase subunit beta-2 [Plutella xylostella]	Manduca and Plutella	2	18	2	9.31
1714784318	ATP synthase lipid-binding protein, mitochondrial [Manduca sexta]	Manduca	1	39	1	8.76
768439318	V-type proton ATPase 116 kDa subunit a isoform 1 isoform X3 [Plutella xylostella]	Plutella	12	60	2	8.13
768415447	calcium-transporting ATPase sarcoplasmic/endoplasmic reticulum type isoform X1 [Plutella xylostella]	Plutella	21	229	2	7.81
1714824983	synaptic vesicle glycoprotein 2C-like isoform X1 [Manduca sexta]	Manduca	1	6	1	7.70
1714843767	protein transport protein Sec61 subunit alpha [Manduca sexta]	Manduca	9	68	9	7.44
1714842681	putative pre-mRNA-splicing factor ATP dependent RNA helicase PRP1 [Manduca sexta]	Manduca	6	18	6	7.34
768410384	translational activator GCN1-like [Plutella xylostella]	Plutella	5	16	5	7.29
768431223	LOW QUALITY PROTEIN: interleukin enhancer-binding factor 2 homolog [Plutella xylostella]	Plutella	3	10	2	7.12
1714837734	innexin inx2 [Manduca sexta]	Manduca	2	8	2	6.99
768414774	V-type proton ATPase subunit H isoform X1 [Plutella xylostella]	Plutella	16	92	2	6.43

1714829531	dolichyl-diphosphooligosaccharide--protein glycosyltransferase subunit STT3A [Manduca sexta]	Manduca	4	13	4	6.23
768413506	neurochondrin homolog [Plutella xylostella]	Plutella	5	23	1	6.01
1714820720	vacuolar protein sorting-associated protein 35 isoform X1 [Manduca sexta]	Manduca	2	9	2	5.79
1127252922	UDP-glycosyltransferase-42F1 [Plutella xylostella]	Plutella	1	3	1	5.75
1714843905	lysine--tRNA ligase isoform X1 [Manduca sexta]	Manduca	5	40	2	5.59
768429978	programmed cell death protein 6 isoform X1 [Plutella xylostella]	Plutella	3	13	3	5.47
165932398	cytochrome c oxidase subunit II (mitochondrion) [Manduca sexta]	Manduca	5	35	1	5.46
1714839839	UDP-glucuronosyltransferase 2B4-like [Manduca sexta]	Manduca	2	10	1	5.38
1714847100	UDP-glucuronosyltransferase 2B4-like isoform X1 [Manduca sexta]	Manduca	3	12	2	5.35
768444918	clustered mitochondria protein homolog [Plutella xylostella]	Plutella	7	19	2	5.32
768408066	hexokinase type 2 isoform X1 [Plutella xylostella]	Plutella	10	32	2	5.30
1714825426	importin-5 [Manduca sexta]	Manduca	9	51	9	5.28
1714818289	synaptojanin-1 [Manduca sexta]	Manduca	1	6	1	5.17
768442606	vesicle-trafficking protein SEC22b [Plutella xylostella]	Plutella	4	9	4	5.11
768421373	glutamate synthase [NADH], amyloplastic like [Plutella xylostella]	Plutella	7	20	3	5.05
768442023	ATP-dependent RNA helicase WM6 [Plutella xylostella]	Plutella	12	53	1	4.85

Accession no.	Protein	Organism	# Pept		# Unique Peptides	Fold-change difference
1714817478	translocon-associated protein subunit gamma [Manduca sexta]	Manduca	2	19	2	4.85
1714787824	Krueppel homolog 2 [Manduca sexta]	Manduca	1	7	1	4.76
1714802768	V-type proton ATPase 16 kDa proteolipid subunit [Manduca sexta]	Manduca	2	36	2	4.76
768418561	vacuolar protein sorting-associated	Plutella	2	10	2	4.74

	protein 29 [Plutella xylostella]						
1714854095	phosphate carrier protein, mitochondrial like [Manduca sexta]	Manduca	6	53	1	4.49
768416141	zinc finger protein 816-like [Plutella xylostella]	Plutella	1	4	1	4.36
1714838480	metal transporter CNNM4-like [Manduca sexta]	Manduca	5	27	5	4.29
768416529	tryptophan 2,3-dioxygenase-like [Plutella xylostella]	Plutella	3	15	3	4.26
1714824089	piwi-like protein Siwi [Manduca sexta]	Manduca	7	24	5	4.12
1714833646	synaptotagmin 1 isoform X1 [Manduca sexta]	Manduca	2	5	2	3.99
768422560	pre-mRNA-processing-splicing factor 8 [Plutella xylostella]	Manduca and Plutella	6	12	6	3.97
1714854061	nicotinate phosphoribosyltransferase isoform X1 [Manduca sexta]	Manduca	1	9	1	3.92
1714821278	thioredoxin reductase-like selenoprotein T homolog CG3887 [Manduca sexta]	Manduca	2	17	2	3.92
1714835193	splicing factor 3B subunit 1 [Manduca sexta]	Manduca	3	5	3	3.92
768448059	coatomer subunit beta'-like [Plutella xylostella]	Plutella	5	17	3	3.91
768419379	muscle M-line assembly protein unc-89- like [Plutella xylostella]	Plutella	9	42	4	3.90
1714790939	coatomer subunit gamma [Manduca sexta]	Manduca	6	9	4	3.87
768415371	sodium/potassium-transporting ATPase subunit alpha isoform X1 [Plutella xylostella]	Plutella	29	148	29	3.85
768442347	probable N-acetyltransferase san [Plutella xylostella]	Plutella	2	9	1	3.83
1714858387	ADP,ATP carrier protein [Manduca sexta]	Manduca	16	204	2	3.82
488444153	serine/threonine-protein phosphatase 2A catalytic subunit [Plutella xylostella]	Manduca and Plutella	5	26	5	3.78
1714824281	1,4-alpha-glucan-branching enzyme [Manduca sexta]	Manduca	5	22	2	3.77
1714806575	eukaryotic translation initiation factor 4 gamma 3-like isoform X1 [Manduca sexta]	Manduca	3	10	3	3.76
1714845569	eukaryotic translation initiation factor 3 subunit K [Manduca sexta]	Manduca	7	26	6	3.73

768422071	plastin-3 isoform X3 [Plutella xylostella]	Manduca and Plutella	8	38	7	3.71
768443734	phosphoribosylformylglycinamidine synthase [Plutella xylostella]	Plutella	7	64	3	3.70
1714835219	serine/threonine-protein phosphatase 2B catalytic subunit 3-like [Manduca sexta]	Manduca	10	25	10	3.67
1714819420	D-beta-hydroxybutyrate dehydrogenase, mitochondrial [Manduca sexta]	Manduca	6	46	2	3.64
1714836738	V-type proton ATPase subunit d [Manduca sexta]	Manduca	5	18	2	3.60
768449720	coatomer subunit beta-like [Plutella xylostella]	Plutella	5	16	4	3.59
1714858948	dolichyl-diphosphooligosaccharide-- protein glycosyltransferase subunit 1, partial [Manduca sexta]	Manduca	6	48	6	3.59
768424392	probable citrate synthase 2, mitochondrial [Plutella xylostella]	Plutella	7	57	4	3.55

Accession no.	Protein	Organism	# Pept		# Unique Peptides	Fold-change difference
1714836771	aromatic-L-amino-acid decarboxylase [Manduca sexta]	Manduca	2	12	1	3.54
768421871	cell differentiation protein RCD1 homolog [Plutella xylostella]	Plutella	1	1	1	3.54
1714815611	dipeptidyl peptidase 3 isoform X1 [Manduca sexta]	Manduca	3	25	1	3.51
768445010	clathrin heavy chain isoform X1 [Plutella xylostella]	Plutella	33	177	3	3.46
1714796863	calcium-binding mitochondrial carrier protein Aralar1 isoform X1 [Manduca sexta]	Manduca	7	40	5	3.44
1714833962	aspartate aminotransferase, cytoplasmic [Manduca sexta]	Manduca	3	33	3	3.43
1714794110	puromycin-sensitive aminopeptidase [Manduca sexta]	Manduca	10	40	6	3.42
787071490	adenine nucleotide translocase insect3 [Plutella xylostella]	Plutella	16	203	2	3.37
768428328	puromycin-sensitive aminopeptidase isoform X1 [Plutella xylostella]	Plutella	6	30	2	3.34

768430159	arginine--tRNA ligase, cytoplasmic [Plutella xylostella]	Plutella	4	20	1	3.33
768441273	scavenger receptor class B member 1 isoform X1 [Plutella xylostella]	Manduca and Plutella	2	7	2	3.31
1714787675	amino acid transporter AVT1B-like [Manduca sexta]	Manduca	1	7	1	3.30
768407834	transmembrane emp24 domain-containing protein bai [Plutella xylostella]	Plutella	4	36	4	3.29
1714812709	coatomer subunit alpha isoform X2 [Manduca sexta]	Manduca	14	28	3	3.23
768407893	presequence protease, mitochondrial-like [Plutella xylostella]	Plutella	3	9	2	3.15
768436859	kynurenine 3-monooxygenase-like [Plutella xylostella]	Plutella	2	6	2	3.14
768427005	uncharacterized protein LOC105385824 [Plutella xylostella]	Plutella	5	21	2	3.10
1714816610	glutamine--fructose-6-phosphate aminotransferase [isomerizing] 1-like [Manduca sexta]	Manduca	4	14	4	3.08
1714845899	proline dehydrogenase 1, mitochondrial like [Manduca sexta]	Manduca	5	44	5	3.07
1714797181	40S ribosomal protein SA [Manduca sexta]	Manduca	10	118	2	3.07
1714779221	N-alpha-acetyltransferase 15, NatA auxiliary subunit [Manduca sexta]	Manduca	4	11	4	3.07
1714779918	casein kinase II subunit alpha isoform X1 [Manduca sexta]	Manduca	8	17	8	3.07
768446211	ATP-dependent RNA helicase Ddx1-like [Plutella xylostella]	Plutella	2	7	2	3.06
768428731	uncharacterized protein LOC105386610 [Plutella xylostella]	Manduca and Plutella	1	10	1	3.04
768409771	V-type proton ATPase 116 kDa subunit a isoform 1-like isoform X1 [Plutella xylostella]	Plutella	8	56	8	3.03
1714789770	AFG3-like protein 2 [Manduca sexta]	Manduca	13	41	3	3.02
1714780065	translocating chain-associated membrane protein 1 [Manduca sexta]	Manduca	1	9	1	3.00
1714798516	uncharacterized protein LOC115441016 [Manduca sexta]	Manduca	13	96	13	2.97
768440535	laminin subunit alpha-like, partial [Plutella xylostella]	Plutella	6	30	2	2.96
1714827818	myosin heavy chain, muscle isoform	#N/A	169	2916	0	2.95

	X14 [Manduca sexta]					
1714816500	26S proteasome non-ATPase regulatory subunit 6 [Manduca sexta]	Manduca	5	27	3	2.94
1714830307	threonine--tRNA ligase, cytoplasmic isoform X1 [Manduca sexta]	Manduca	11	52	11	2.92

**Accession no.**	**Protein**	**Organism**	**# Pept**		**# Unique Peptides**	**Fold-change difference**
1714797205	ubiquitin carboxyl-terminal hydrolase 7- like isoform X1 [Manduca sexta]	Manduca	11	50	7	2.92
1714790982	NADH-ubiquinone oxidoreductase 49 kDa subunit [Manduca sexta]	Manduca	8	45	8	2.92
1714784806	LOW QUALITY PROTEIN: tripeptidyl peptidase 2 [Manduca sexta]	Manduca	2	13	2	2.91
1714844215	tubulin beta chain-like [Manduca sexta]	Manduca	14	118	3	2.91
768438622	calcium/calmodulin-dependent protein kinase type II alpha chain [Plutella xylostella]	Plutella	5	26	1	2.91
1714795867	xanthine dehydrogenase [Manduca sexta]	Manduca	4	20	4	2.90
1714851726	protein krasavietz [Manduca sexta]	Manduca	5	14	5	2.89
1714786476	26S proteasome non-ATPase regulatory subunit 12 [Manduca sexta]	Manduca	7	38	7	2.88
768426480	serine/threonine-protein phosphatase PP2A 65 kDa regulatory subunit [Plutella xylostella]	Plutella	11	64	1	2.88
1714808308	clathrin heavy chain [Manduca sexta]	Manduca	40	202	10	2.86
768433865	eukaryotic translation initiation factor 3 subunit B [Plutella xylostella]	Plutella	12	46	12	2.85
1714804170	calcium-transporting ATPase sarcoplasmic/endoplasmic reticulum type isoform X1 [Manduca sexta]	Manduca	26	266	7	2.82
1714812923	NADP-dependent malic enzyme-like isoform X1 [Manduca sexta]	Manduca	10	58	10	2.81
1714830135	probable aminopeptidase NPEPL1 isoform X1 [Manduca sexta]	Manduca	2	11	2	2.79
1714798431	uncharacterized protein	Manduca	11	102	3	2.76

	LOC115440997 isoform X1 [Manduca sexta]					
768429249	LOW QUALITY PROTEIN: 26S proteasome non-ATPase regulatory subunit 1-like [Plutella xylostella]	Plutella	12	64	2	2.72
768439997	lysine--tRNA ligase isoform X1 [Plutella xylostella]	Plutella	5	46	2	2.71
1714805039	dolichyl-diphosphooligosaccharide-- protein glycosyltransferase 48 kDa subunit [Manduca sexta]	Manduca	5	30	5	2.70
1714844507	endocuticle structural glycoprotein SgAbd 8-like [Manduca sexta]	Manduca	3	7	3	2.67
768433761	uncharacterized protein C05D11.1-like [Plutella xylostella]	Plutella	2	9	2	2.66
1714834105	juvenile hormone epoxide hydrolase-like [Manduca sexta]	Manduca	1	8	1	2.63
768429473	D-beta-hydroxybutyrate dehydrogenase, mitochondrial isoform X1 [Plutella xylostella]	Plutella	5	27	1	2.62
1714841381	ADP-ribosylation factor-like protein 8 [Manduca sexta]	Manduca	3	7	3	2.61
1714816572	glutamate--cysteine ligase catalytic subunit [Manduca sexta]	Manduca	2	10	2	2.59
1714836753	trifunctional enzyme subunit alpha, mitochondrial [Manduca sexta]	Manduca	5	43	1	2.58
1714832549	LOW QUALITY PROTEIN: multidrug resistance protein homolog 49-like [Manduca sexta]	Manduca	3	8	3	2.57
1714839511	putative tricarboxylate transport protein, mitochondrial [Manduca sexta]	Manduca	5	32	5	2.57
768448701	uncharacterized protein LOC105396107 [Plutella xylostella]	Plutella	1	1	1	2.55
1714817988	cytochrome c oxidase subunit 4 isoform 1, mitochondrial-like [Manduca sexta]	Manduca	2	12	2	2.54
1714823704	dolichyl-diphosphooligosaccharid e-- protein glycosyltransferase subunit 2 [Manduca sexta]	Manduca	2	10	2	2.54
768411720	2-oxoglutarate dehydrogenase, mitochondrial [Plutella xylostella]	Plutella	28	289	1	2.53

**Accession no.**	**Protein**	**Organism**	**# Pept**		**# Unique Peptides**	**Fold-cha nge differen**

						ce
1714795408	cAMP-dependent protein kinase catalytic subunit 1 [Manduca sexta]	Manduca	7	19	7	2.50
768443470	purine nucleoside phosphorylase-like isoform X1 [Plutella xylostella]	Manduca and Plutella	1	10	1	2.47
768424601	ATP-binding cassette sub-family G member 1-like [Plutella xylostella]	Manduca and Plutella	1	9	1	2.47
1714833749	glycogen phosphorylase [Manduca sexta]	Manduca	23	162	14	2.47
1714798768	phosphoribosylformylglycinamidine synthase [Manduca sexta]	Manduca	10	103	6	2.45
1714816107	myrosinase 1-like [Manduca sexta]	Manduca	1	5	1	2.44
1714841515	reticulon-1 isoform X1 [Manduca sexta]	Manduca	2	9	1	2.44
1714836369	ATP-citrate synthase [Manduca sexta]	Manduca	39	325	14	2.43
1714795342	mitochondrial proton/calcium exchanger protein [Manduca sexta]	Manduca	7	33	7	2.42
768421658	guanine nucleotide-binding protein G(o) subunit alpha isoform X1 [Plutella xylostella]	Manduca and Plutella	8	34	8	2.42
1714828155	ras-related protein Ral-a isoform X1 [Manduca sexta]	Manduca	1	1	1	2.40
768441490	cytosolic purine 5'-nucleotidase isoform X1 [Plutella xylostella]	Plutella	4	43	4	2.39
1714837777	116 kDa U5 small nuclear ribonucleoprotein component [Manduca sexta]	Manduca	5	10	5	2.38
768446145	FUN14 domain-containing protein 1-like isoform X1 [Plutella xylostella]	Manduca and Plutella	1	4	1	2.37
768439586	calcium-binding mitochondrial carrier protein Aralar1-like [Plutella xylostella]	Plutella	3	23	1	2.36
768432945	extended synaptotagmin-2-A-like, partial [Plutella xylostella]	Plutella	8	27	2	2.34
768413292	mitochondrial-processing peptidase subunit alpha [Plutella xylostella]	Plutella	2	7	1	2.34
1714785566	atlastin isoform X1 [Manduca sexta]	Manduca	2	4	2	2.32
1714786519	alpha-actinin, sarcomeric isoform X1 [Manduca sexta]	Manduca	61	664	16	2.32
1714796960	tolloid-like protein 1 [Manduca sexta]	Manduca	1	8	1	2.31
768408615	alpha-actinin, sarcomeric-like [Plutella xylostella]	Plutella	50	555	5	2.29

1714789907	plasma membrane calcium-transporting ATPase 2 isoform X1 [Manduca sexta]	Manduca	25	139	25	2.29
1714788345	ATP-dependent RNA helicase WM6 [Manduca sexta]	Manduca	18	88	7	2.29
987437870	60 kDa heat shock protein, mitochondrial like [Plutella xylostella]	Plutella	18	243	15	2.27
768426118	regulator of nonsense transcripts 1-like [Plutella xylostella]	Manduca and Plutella	5	13	5	2.27
768425216	eukaryotic translation initiation factor 3 subunit K [Plutella xylostella]	Plutella	4	9	3	2.27
768414045	S-formylglutathione hydrolase [Plutella xylostella]	Plutella	3	25	3	2.26
768419808	NADP-dependent malic enzyme isoform X1 [Plutella xylostella]	Plutella	8	43	3	2.25
1714836224	ATP synthase subunit gamma, mitochondrial [Manduca sexta]	Manduca	3	40	2	2.25
671871149	P450 CYP6 family protein 6 [Plutella xylostella]	Plutella	1	11	1	2.23
768433478	myosin heavy chain, muscle isoform X15 [Plutella xylostella]	Plutella	158	2787	2	2.22
1714807772	integrin beta-PS [Manduca sexta]	Manduca	2	6	2	2.22
768410192	UDP-glucose:glycoprotein glucosyltransferase [Plutella xylostella]	Plutella	3	14	1	2.22
768443210	tripeptidyl-peptidase 2 [Plutella xylostella]	Plutella	2	6	2	2.21
1714782743	long-chain-fatty-acid--CoA ligase 4 isoform X1 [Manduca sexta]	Manduca	4	40	2	2.21
768423690	bifunctional glutamate/proline--tRNA ligase isoform X1 [Plutella xylostella]	Plutella	7	34	6	2.19

Accession no.	Protein	Organism	# Pept		# Unique Peptides	Fold-change difference
1714853725	L-asparaginase-like [Manduca sexta]	Manduca	3	8	3	2.19
768451820	transaldolase-like [Plutella xylostella]	Plutella	3	13	2	2.19
1714786893	protein arginine N-methyltransferase 1 [Manduca sexta]	Manduca	4	21	4	2.15
768426977	carnitine O-palmitoyltransferase 2, mitochondrial-like [Plutella xylostella]	Plutella	1	2	1	2.15

1127252882	Fatty acid synthase-4, partial [Plutella xylostella]	Plutella	3	35	1	2.14
768443446	uncharacterized protein K02A2.6-like [Plutella xylostella]	Plutella	1	1	1	2.13
1714818345	alpha-aminoadipic semialdehyde synthase, mitochondrial [Manduca sexta]	Manduca	12	80	3	2.13
1714817409	26S proteasome non-ATPase regulatory subunit 2 [Manduca sexta]	Manduca	14	98	4	2.13
1714823275	putative ATP synthase subunit f, mitochondrial [Manduca sexta]	Manduca	1	18	1	2.13
164683438	eukaryotic initiation factor 4A [Plutella xylostella]	Plutella	19	175	3	2.13
1714814714	uncharacterized protein LOC115444704, partial [Manduca sexta]	Manduca	51	186	34	2.12
768424454	V-type proton ATPase subunit C isoform X1 [Plutella xylostella]	Plutella	11	77	3	2.12
671871185	P450 CYP6 family protein 10 [Plutella xylostella]	Plutella	2	10	2	2.11
768438040	ATP-binding cassette sub-family E member 1 [Plutella xylostella]	Plutella	8	44	8	2.10
768443222	proteasome activator complex subunit 3 isoform X1 [Plutella xylostella]	Plutella	4	13	4	2.10
117970173	pxS-adenosyl-L-homocysteine hydrolase [Plutella xylostella]	Plutella	13	137	2	2.10
768433673	C-terminal-binding protein [Plutella xylostella]	Plutella	7	26	7	2.09
1714847252	serine/threonine-protein phosphatase 2A 65 kDa regulatory subunit A alpha isoform [Manduca sexta]	Manduca	14	82	4	2.09
1714822723	acetyl-CoA carboxylase isoform X1 [Manduca sexta]	Manduca	34	186	19	2.09
768445239	T-complex protein 1 subunit alpha [Plutella xylostella]	Plutella	13	101	1	2.09
1714837691	mitochondrial import receptor subunit TOM40 homolog 1-like [Manduca sexta]	Manduca	4	38	4	2.08
1714813192	ATP synthase subunit b, mitochondrial [Manduca sexta]	Manduca	1	14	1	2.08
768410047	uncharacterized protein LOC105387140 isoform X1 [Plutella xylostella]	Plutella	10	100	2	2.07
1127252884	Fatty acid synthase-5 [Plutella xylostella]	Plutella	2	11	1	2.07

768418331	glyoxylate reductase/hydroxypyruvate reductase-like [Plutella xylostella]	Plutella	1	4	1	2.07
1714806071	dihydropyrimidine dehydrogenase [NADP(+)] [Manduca sexta]	Manduca	11	46	11	2.07
1714781838	venom carboxylesterase-6-like [Manduca sexta]	Manduca	1	6	1	2.06
768433466	myosin heavy chain, muscle isoform X9 [Plutella xylostella]	Plutella	160	2817	1	2.06
768438558	glucose-6-phosphate isomerase [Plutella xylostella]	Plutella	6	68	2	2.06
768419683	adipocyte plasma membrane-associated protein isoform X1 [Plutella xylostella]	Plutella	1	7	1	2.05
768425765	T-complex protein 1 subunit beta-like [Plutella xylostella]	Plutella	10	56	7	2.05
768450471	uncharacterized protein LOC105397069 [Plutella xylostella]	Plutella	1	9	1	2.04
768430234	importin subunit beta-1-like [Plutella xylostella]	Plutella	2	15	2	2.04

Accession no.	Protein	Organism	# Pept		# Unique Peptides	Fold-change difference
1714848642	pentatricopeptide repeat-containing protein 2, mitochondrial-like [Manduca sexta]	Manduca	1	8	1	2.02
1714851519	V-type proton ATPase 116 kDa subunit a isoform X3 [Manduca sexta]	Manduca	13	63	3	2.02
768424460	ATP synthase subunit gamma, mitochondrial [Plutella xylostella]	Plutella	4	29	3	2.02
1714815555	ruvB-like helicase 1 [Manduca sexta]	Manduca	6	29	6	2.01
1714856098	H/ACA ribonucleoprotein complex subunit 4-like [Manduca sexta]	Manduca	10	63	10	2.01
1714843560	cytochrome P450 9e2-like [Manduca sexta]	Manduca	3	19	3	2.00
1714799935	adenylate kinase [Manduca sexta]	Manduca	8	64	6	0.50
1714821271	COP9 signalosome complex subunit 9 [Manduca sexta]	Manduca	1	6	1	0.50
768433032	CCHC-type zinc finger protein CG3800 [Plutella xylostella]	Plutella	2	19	2	0.50

1714812765	probable small nuclear ribonucleoprotein Sm D2 [Manduca sexta]	Manduca	7	51	7	0.50
1714819213	heterogeneous nuclear ribonucleoprotein A1-like [Manduca sexta]	Manduca	3	23	3	0.50
1714815507	PDZ and LIM domain protein Zasp isoform X6 [Manduca sexta]	Manduca	11	94	8	0.49
1714832101	12 kDa FK506-binding protein-like [Manduca sexta]	Manduca	3	16	3	0.49
1714843374	tropomyosin-2 isoform X13 [Manduca sexta]	Manduca	39	739	8	0.49
1714780041	probable elongation factor 1-delta isoform X1 [Manduca sexta]	Manduca	1	13	1	0.49
768437513	protein held out wings [Plutella xylostella]	Plutella	3	6	3	0.49
768443164	flavin reductase (NADPH) [Plutella xylostella]	Plutella	1	12	1	0.49
1714838106	uncharacterized protein LOC115449737 [Manduca sexta]	Manduca	1	9	1	0.49
1714814099	40S ribosomal protein S20 [Manduca sexta]	Manduca	3	26	3	0.49
770075562	cytochrome c [Plutella xylostella]	Plutella	4	46	4	0.49
1714852094	uncharacterized protein LOC115453316 [Manduca sexta]	Manduca	1	16	1	0.49
1714824563	alpha-catulin isoform X1 [Manduca sexta]	Manduca	1	9	1	0.49
1714808844	ribosome-binding protein 1 isoform X1 [Manduca sexta]	Manduca	3	11	3	0.49
1714852366	uncharacterized protein LOC115455253 [Manduca sexta]	Manduca	1	9	1	0.48
768447608	bifunctional 3'-phosphoadenosine 5'-phosphosulfate synthase [Plutella xylostella]	Plutella	8	42	4	0.48
1714800027	chromobox protein homolog 1-like isoform X1 [Manduca sexta]	Manduca	3	12	3	0.48
1714794276	heterogeneous nuclear ribonucleoprotein M isoform X1 [Manduca sexta]	Manduca	6	39	6	0.48
1714837793	endoplasmic reticulum chaperone BiP isoform X2 [Manduca sexta]	Manduca	32	494	4	0.48
768447669	phosphoglycerate mutase 1-like [Plutella xylostella]	Plutella	7	81	7	0.48
1714828204	titin-like isoform X1 [Manduca sexta]	Manduca	2	5	2	0.48

768425048	heterogeneous nuclear ribonucleoprotein 87F-like [Plutella xylostella]	Plutella	7	66	5	0.48
1714846630	aldo-keto reductase family 1 member B1- like [Manduca sexta]	Manduca	5	51	3	0.47
1714787004	eukaryotic translation initiation factor 1A, X-chromosomal [Manduca sexta]	Manduca	5	13	5	0.47

**Accession no.**	**Protein**	**Organism**	**# Pept**		**# Unique Peptides**	**Fold-change difference**
1714817426	enolase [Manduca sexta]	Manduca	11	210	11	0.47
49532856	Ribosomal protein L36A [Plutella xylostella]	Plutella	6	73	6	0.47
1714782021	26S proteasome regulatory subunit 6B [Manduca sexta]	Manduca	21	133	21	0.47
768436047	aldose reductase-like [Plutella xylostella]	Plutella	2	16	1	0.47
1714800745	protein disulfide-isomerase A3 [Manduca sexta]	Manduca	3	32	2	0.47
768423908	uncharacterized protein LOC105384358 isoform X1 [Plutella xylostella]	Plutella	8	88	1	0.47
768430181	putative RNA-binding protein 15 [Plutella xylostella]	Plutella	4	14	4	0.47
1714804470	ubiquitin-fold modifier 1 isoform X2 [Manduca sexta]	Manduca	4	26	4	0.47
1714842438	ubiquitin-conjugating enzyme E2 G1 isoform X1 [Manduca sexta]	Manduca	2	8	2	0.46
768412058	cytochrome b-c1 complex subunit 7-like [Plutella xylostella]	Plutella	1	9	1	0.46
1714830944	UV excision repair protein RAD23 homolog A [Manduca sexta]	Manduca	5	20	5	0.46
1714843403	tropomyosin-1 [Manduca sexta]	Manduca	29	672	27	0.46
1714819169	heterogeneous nuclear ribonucleoprotein 87F-like [Manduca sexta]	Manduca	4	35	2	0.46
1714833115	aldo-keto reductase AKR2E4-like isoform X1 [Manduca sexta]	Manduca	2	10	2	0.46
768420291	neprilysin-11 isoform X1 [Plutella xylostella]	Plutella	2	5	2	0.46
1714808547	LOW QUALITY PROTEIN: serpin A9-like [Manduca sexta]	Manduca	1	22	1	0.46

1714820847	KH domain-containing, RNA-binding, signal transduction-associated protein 2-like [Manduca sexta]	Manduca	2	14	2	0.46
1714810293	heat shock factor-binding protein 1 isoform X1 [Manduca sexta]	Manduca	6	14	6	0.46
1714786330	L-xylulose reductase-like [Manduca sexta]	Manduca	5	39	4	0.46
1714793625	ubiquitin domain-containing protein UBFD1-like [Manduca sexta]	Manduca	1	2	1	0.45
768415639	triosephosphate isomerase [Plutella xylostella]	Plutella	6	81	2	0.45
770075529	60S ribosomal protein L31 [Plutella xylostella]	Plutella	6	86	2	0.45
1714823019	enhancer of rudimentary homolog [Manduca sexta]	Manduca	3	14	3	0.45
1714844700	aldo-keto reductase family 1 member B1- like isoform X1 [Manduca sexta]	Manduca	6	72	5	0.45
768447564	26S protease regulatory subunit 6A-B [Plutella xylostella]	Plutella	20	144	2	0.45
1714820445	branched-chain-amino-acid aminotransferase, cytosolic [Manduca sexta]	Manduca	2	5	2	0.45
1714811260	aldehyde dehydrogenase X, mitochondrial like [Manduca sexta]	Manduca	7	62	2	0.45
1714814635	nuclear migration protein nudC [Manduca sexta]	Manduca	5	29	5	0.45
768412076	lamin Dm0-like [Plutella xylostella]	Plutella	21	175	5	0.45
768413904	heat shock 70 kDa protein cognate 3-like [Plutella xylostella]	Plutella	33	480	5	0.45
768408976	protein D2-like isoform X1 [Plutella xylostella]	Plutella	4	27	4	0.44
1714801062	rho GDP-dissociation inhibitor 1 isoform X2 [Manduca sexta]	Manduca	5	33	5	0.44
1714814022	tubulin beta chain-like isoform X2 [Manduca sexta]	Manduca	19	264	7	0.44
1714850531	clathrin light chain-like isoform X1 [Manduca sexta]	Manduca	2	16	2	0.44

**Accession no.**	**Protein**	**Organism**	**# Pept**		**# Unique Peptides**	**Fold-change difference**

1714850878	endothelial differentiation-related factor 1 homolog isoform X1 [Manduca sexta]	Manduca	2	14	2	0.44
1714816672	extended synaptotagmin-2-A isoform X1 [Manduca sexta]	Manduca	9	35	3	0.44
768428604	thioredoxin-like protein 1 [Plutella xylostella]	Plutella	2	22	2	0.44
1714829845	SUMO-conjugating enzyme UBC9-A [Manduca sexta]	Manduca and Plutella	8	32	8	0.44
768421686	protein lethal(2)essential for life-like [Plutella xylostella]	Plutella	3	34	3	0.44
1714856382	NEDD8 [Manduca sexta]	Manduca	3	29	3	0.44
768447461	myosin regulatory light chain 2-like [Plutella xylostella]	Plutella	4	108	4	0.43
1714818754	membrane-bound alkaline phosphatase like [Manduca sexta]	Manduca	1	6	1	0.43
1714819196	prefoldin subunit 6 [Manduca sexta]	Manduca	5	32	5	0.43
768419209	stress-induced-phosphoprotein 1-like [Plutella xylostella]	Plutella	5	28	5	0.43
768420892	thioredoxin reductase 1, mitochondrial like isoform X1 [Plutella xylostella]	Plutella	3	20	1	0.43
1714803661	ras GTPase-activating protein-binding protein 2, partial [Manduca sexta]	Manduca	5	21	2	0.43
1714781802	calmodulin isoform X1 [Manduca sexta]	Manduca	14	159	13	0.43
1714780838	glycine-rich cell wall structural protein [Manduca sexta]	Manduca	2	32	2	0.43
1714812774	serine/threonine-protein phosphatase 2A 55 kDa regulatory subunit B alpha isoform isoform X1 [Manduca sexta]	Manduca	2	8	2	0.42
768411157	classical arabinogalactan protein 4-like [Plutella xylostella]	Plutella	1	8	1	0.42
1714785732	COP9 signalosome complex subunit 1 [Manduca sexta]	Manduca	3	4	3	0.42
1714815968	sialic acid synthase [Manduca sexta]	Manduca	4	21	2	0.42
1714788098	myosin regulatory light chain sqh [Manduca sexta]	Manduca	7	30	7	0.42
768449175	hydroxyacyl-coenzyme A dehydrogenase, mitochondrial-like [Plutella xylostella]	Plutella	5	44	1	0.42
768419247	proteasome subunit alpha type-6-like [Plutella xylostella]	Plutella	6	49	2	0.42
1714783673	uncharacterized protein	Manduca	1	10	1	0.42

	LOC115445754 isoform X1 [Manduca sexta]					
1714843393	tropomyosin-1, isoforms 9A/A/B isoform X19 [Manduca sexta]	Manduca	22	310	6	0.41
1714793065	ubiquitin carboxyl-terminal hydrolase 5 [Manduca sexta]	Manduca	6	25	5	0.41
768408131	uncharacterized protein ZC395.10-like [Plutella xylostella]	Manduca and Plutella	1	5	1	0.41
768425018	glutaredoxin 3 [Plutella xylostella]	Plutella	2	23	2	0.41
1714800977	histone H3.3 [Manduca sexta]	Manduca and Plutella	5	73	1	0.41
1714843381	tropomyosin-2 isoform X15 [Manduca sexta]	#N/A	24	429	0	0.41
1714844337	proteasome subunit alpha type-6-like [Manduca sexta]	Manduca	7	52	3	0.41
1714822547	hepatocyte growth factor-regulated tyrosine kinase substrate isoform X1 [Manduca sexta]	Manduca	3	11	3	0.41
1714784627	eukaryotic translation initiation factor 3 subunit A [Manduca sexta]	Manduca	1	10	1	0.41
1714800243	neurogenic locus notch homolog protein 1 [Manduca sexta]	Manduca	5	31	4	0.41
768447965	heat shock protein beta-1-like [Plutella xylostella]	Plutella	8	63	8	0.41
768423176	neurogenic locus notch homolog protein 4- like [Plutella xylostella]	Plutella	2	21	1	0.41
1714792933	LOW QUALITY PROTEIN: high mobility group protein D-like [Manduca sexta]	Manduca	1	33	1	0.40

**Accession no.**	**Protein**	**Organism**	**# Pept**		**# Unique Peptides**	**Fold-change difference**
1714846832	selenoprotein M-like [Manduca sexta]	Manduca	1	4	1	0.40
1714789564	U6 snRNA-associated Sm-like protein LSm6 [Manduca sexta]	Manduca	2	9	2	0.40
768420965	peptidyl-prolyl cis-trans isomerase B [Plutella xylostella]	Plutella	4	33	4	0.40
768427950	small nuclear ribonucleoprotein Sm D1 [Plutella xylostella]	Plutella	2	20	1	0.40
1714815438	peroxiredoxin-6 [Manduca sexta]	Manduca	2	8	2	0.40

768420532	tropomyosin-2 isoform X2 [Plutella xylostella]	Plutella	20	368	2	0.39
822092476	glyceraldehyde-3-phosphate dehydrogenase [Plutella xylostella]	Plutella	7	259	1	0.39
1714835189	vigilin isoform X2 [Manduca sexta]	Manduca	15	113	7	0.39
1714830170	triosephosphate isomerase [Manduca sexta]	Manduca	7	93	3	0.39
1714857497	bifunctional 3'-phosphoadenosine 5'-phosphosulfate synthase 2-like [Manduca sexta]	Manduca	5	32	1	0.39
1714799386	protein LSM14 homolog B isoform X1 [Manduca sexta]	Manduca	3	16	3	0.39
1714839079	ferritin subunit [Manduca sexta]	Manduca	1	9	1	0.39
1714790157	protein lethal(2)essential for life-like [Manduca sexta]	Manduca	6	93	4	0.39
768436581	arginine kinase isoform X1 [Plutella xylostella]	Plutella	21	1095	1	0.39
1714844694	alpha-centractin [Manduca sexta]	Manduca	5	13	5	0.38
1714805288	cytosolic carboxypeptidase 2-like [Manduca sexta]	Manduca	1	6	1	0.38
768445006	dehydrogenase/reductase SDR family member 4 [Plutella xylostella]	Plutella	1	9	1	0.38
1714815957	carbonic anhydrase 1 [Manduca sexta]	Manduca	1	6	1	0.38
768410779	serine/arginine repetitive matrix protein 1- like isoform X1 [Plutella xylostella]	Manduca and Plutella	2	10	2	0.38
1714786277	L-xylulose reductase-like [Manduca sexta]	Manduca	1	6	1	0.38
1714840539	calreticulin [Manduca sexta]	Manduca	8	92	4	0.38
1714851643	S-methyl-5'-thioadenosine phosphorylase like [Manduca sexta]	Manduca	2	7	2	0.38
1714851245	RNA transcription, translation and transport factor protein [Manduca sexta]	Manduca	2	3	2	0.38
768438732	U1 small nuclear ribonucleoprotein C [Plutella xylostella]	Manduca and Plutella	2	9	2	0.38
768415052	mesencephalic astrocyte-derived neurotrophic factor homolog [Plutella xylostella]	Manduca and Plutella	1	3	1	0.37
768445306	aldehyde dehydrogenase X, mitochondrial like [Plutella xylostella]	Plutella	9	77	3	0.37
1714795047	PC4 and SFRS1-interacting protein isoform X1 [Manduca sexta]	Manduca	1	6	1	0.37

1714795060	translation machinery-associated protein 7 homolog isoform X2 [Manduca sexta]	Manduca	3	9	3	0.37
768410802	LOW QUALITY PROTEIN: calumenin-A-like [Plutella xylostella]	Plutella	3	11	2	0.36
1714851484	histone H3-like [Manduca sexta]	Manduca	5	45	1	0.36
768410288	macrophage migration inhibitory factor like [Plutella xylostella]	Plutella	2	21	2	0.36
1714854753	phenoloxidase-activating factor 2-like, partial [Manduca sexta]	Manduca	2	18	2	0.36
768413216	U6 snRNA-associated Sm-like protein LSm3 [Plutella xylostella]	Plutella	1	8	1	0.36
768431908	myotrophin-like [Plutella xylostella]	Plutella	3	10	3	0.36
768425078	transmembrane protease serine 9-like [Plutella xylostella]	Manduca and Plutella	1	5	1	0.36
768447528	sorting nexin-2-like [Plutella xylostella]	Plutella	3	7	2	0.36
768448326	probable prefoldin subunit 4 [Plutella xylostella]	Plutella	1	10	1	0.36

**Accession no.**	**Protein**	**Organism**	**# Pept**		**# Unique Peptides**	**Fold-change difference**
1714851451	striatin isoform X1 [Manduca sexta]	Manduca	3	11	3	0.35
768428921	mRNA-decapping enzyme 1B-like [Plutella xylostella]	Manduca and Plutella	2	7	2	0.35
1714812652	pleckstrin homology domain-containing family F member 2 isoform X1 [Manduca sexta]	Manduca	2	4	2	0.35
1714830106	digestive cysteine proteinase 1 [Manduca sexta]	Manduca	3	14	1	0.35
1714847321	myosin-2 essential light chain isoform X1 [Manduca sexta]	Manduca	5	31	5	0.35
768445694	uncharacterized protein LOC105394551 [Plutella xylostella]	Plutella	2	8	2	0.35
1714799300	chitinase-like protein EN03 isoform X1	Manduca	4	186	4	0.35
1714802054	glutathione S-transferase 2-like isoform X1 [Manduca sexta]	Manduca	1	6	1	0.35
768443011	ubiquitin-fold modifier-conjugating enzyme 1 [Plutella xylostella]	Plutella	3	14	3	0.34
1714853512	26S proteasome regulatory subunit 4 [Manduca sexta]	Manduca	18	81	17	0.34

1714822245	uncharacterized protein LOC115446254 [Manduca sexta]	Manduca	3	13	3	0.34
1714813038	synapse-associated protein of 47 kDa isoform X1 [Manduca sexta]	Manduca	2	6	2	0.33
768414627	putative neuropeptide precursor protein isoform X1 [Plutella xylostella]	Plutella	4	10	4	0.33
1714796329	zinc-type alcohol dehydrogenase-like protein C1773.06c [Manduca sexta]	Manduca	1	4	1	0.33
1714819426	prefoldin subunit 3 [Manduca sexta]	Manduca	2	7	2	0.33
1025716978	thioredoxin-like protein [Plutella xylostella]	Plutella	2	100	2	0.33
1714802920	glycine N-methyltransferase isoform X1 [Manduca sexta]	Manduca	4	38	4	0.33
768418023	ATP-dependent RNA helicase dbp4-like [Plutella xylostella]	Manduca and Plutella	1	8	1	0.32
1714843350	tropomyosin-2 isoform X7 [Manduca sexta]	#N/A	29	533	0	0.32
1714832438	28S ribosomal protein S17, mitochondrial [Manduca sexta]	Manduca	2	6	2	0.32
768419735	troponin C, isoform 3-like [Plutella xylostella]	Plutella	9	118	4	0.32
1714834544	apolipophorin-3 [Manduca sexta]	Manduca	1	27	1	0.32
768412686	microtubule-associated protein RP/EB family member 3, partial [Plutella xylostella]	Plutella	3	27	2	0.31
49532918	cellular retinoic acid binding protein [Plutella xylostella]	Plutella	5	86	4	0.31
1714845982	general transcription factor IIF subunit 1- like [Manduca sexta]	Manduca	4	23	4	0.31
1714795275	transferrin [Manduca sexta]	Manduca	5	31	5	0.31
1714824465	uncharacterized protein LOC115446757 [Manduca sexta]	Manduca	1	5	1	0.30
1714818527	adenylyl cyclase-associated protein 1 isoform X1 [Manduca sexta]	Manduca	4	22	4	0.30
768415969	carbonic anhydrase 15-like [Plutella xylostella]	Plutella	1	3	1	0.30
768421038	vacuolar protein sorting-associated protein VTA1 homolog isoform X1 [Plutella xylostella]	Plutella	1	1	1	0.30
768440608	WW domain-binding protein 2 [Plutella xylostella]	Plutella	1	3	1	0.30
1714853717	U6 snRNA-associated Sm-like protein	Manduca	2	6	2	0.30

	LSm4 [Manduca sexta]					
388252732	beta-1,3-glucan binding protein [Plutella xylostella]	Plutella	1	6	1	0.30
768418109	LDLR chaperone boca [Plutella xylostella]	Plutella	3	19	3	0.29
768431340	SPARC [Plutella xylostella]	Plutella	1	3	1	0.28

**Accession no.**	**Protein**	**Organism**	**# Pept**		**# Unique Peptides**	**Fold-change difference**
768451365	LOW QUALITY PROTEIN: alpha-2-macroglobulin receptor-associated protein like [Plutella xylostella]	Plutella	1	4	1	0.28
768431856	kinesin light chain-like [Plutella xylostella]	Plutella	3	7	2	0.28
1714788570	FK506-binding protein 2 [Manduca sexta]	Manduca	9	59	1	0.28
1714853488	signal recognition particle 54 kDa protein [Manduca sexta]	Manduca	5	25	5	0.28
1714825657	stromal cell-derived factor 2 [Manduca sexta]	Manduca	1	6	1	0.28
1714808375	alpha-tocopherol transfer protein-like [Manduca sexta]	Manduca	2	2	2	0.28
768431748	heterogeneous nuclear ribonucleoprotein K [Plutella xylostella]	Manduca and Plutella	2	10	2	0.28
768439126	transitional endoplasmic reticulum ATPase TER94 [Plutella xylostella]	Plutella	42	372	1	0.28
1714808268	sodium/calcium exchanger regulatory protein 1 [Manduca sexta]	Manduca	2	36	1	0.28
768445580	prefoldin subunit 2 [Plutella xylostella]	Plutella	1	8	1	0.27
768424642	chitooligosaccharidolytic beta-N acetylglucosaminidase [Plutella xylostella]	Plutella	1	2	1	0.27
768439252	troponin T, skeletal muscle isoform X1 [Plutella xylostella]	Plutella	14	160	3	0.27
1127253092	Aldehyde reductase-1 [Plutella xylostella]	Plutella	1	7	1	0.26
768439087	tumor susceptibility gene 101 protein [Plutella xylostella]	Plutella	4	20	4	0.26
1714823349	uncharacterized protein LOC115446507 [Manduca sexta]	Manduca	1	15	1	0.25

768411909	protein dpy-30 homolog [Plutella xylostella]	Manduca and Plutella	1	14	1	0.25
1714848962	charged multivesicular body protein 4b like [Manduca sexta]	Manduca	3	29	3	0.25
768424285	malignant T-cell-amplified sequence 1 homolog [Plutella xylostella]	Plutella	2	11	2	0.24
1714785891	paxillin isoform X1 [Manduca sexta]	Manduca	3	36	1	0.24
1714812937	vacuolar protein sorting-associated protein 4B [Manduca sexta]	Manduca	6	17	5	0.24
768434562	tumor protein D52 isoform X1 [Plutella xylostella]	Plutella	3	24	2	0.24
768419118	glutenin, high molecular weight subunit 12-like [Plutella xylostella]	Plutella	3	37	3	0.24
1033208529	prophenoloxidase 1 [Plutella xylostella]	Plutella	4	27	2	0.24
1714811156	myophilin [Manduca sexta]	Manduca	2	29	2	0.23
1714842649	GILT-like protein 1 isoform X1 [Manduca sexta]	Manduca	1	1	1	0.23
1714792353	uncharacterized protein LOC115456444 [Manduca sexta]	Manduca	2	5	2	0.23
1714851006	protein DDI1 homolog 2-like isoform X1 [Manduca sexta]	Manduca	1	2	1	0.22
1714802943	troponin T, skeletal muscle isoform X1 [Manduca sexta]	Manduca	19	211	8	0.21
1714793781	target of Myb protein 1 isoform X1 [Manduca sexta]	Manduca	3	6	3	0.21
1714822508	protein yellow-like [Manduca sexta]	Manduca	2	4	2	0.21
768439019	proline synthase co-transcribed bacterial homolog protein [Plutella xylostella]	Plutella	1	2	1	0.21
1714832155	protein DEK [Manduca sexta]	Manduca	4	12	4	0.21
1714812934	U6 snRNA-associated Sm-like protein LSm8 [Manduca sexta]	Manduca	2	4	2	0.20
768419725	LOW QUALITY PROTEIN: rap guanine nucleotide exchange factor 2-like [Plutella xylostella]	Plutella	1	2	1	0.19
1714820485	S-phase kinase-associated protein 1 [Manduca sexta]	Manduca	5	23	5	0.19

**Accession no.**	**Protein**	**Organism**	**# Pept**		**# Unique Peptides**	**Fold-change difference**

1714818997	uncharacterized protein LOC115445531 [Manduca sexta]	Manduca	4	40	1	0.19
1714804609	charged multivesicular body protein 2a [Manduca sexta]	Manduca	2	6	2	0.19
1714810464	uncharacterized protein LOC115443743 isoform X1 [Manduca sexta]	Manduca	4	35	1	0.18
1714824104	hsc70-interacting protein-like [Manduca sexta]	Manduca	1	6	1	0.17
768409364	transcription elongation factor B polypeptide 2 [Plutella xylostella]	Plutella	2	5	1	0.17
768444256	barrier-to-autointegration factor B [Plutella xylostella]	Plutella	3	15	3	0.17
1714844440	ELAV-like protein 1 [Manduca sexta]	Manduca	4	23	4	0.17
1714803042	sorting nexin-12 [Manduca sexta]	Manduca	5	22	5	0.16
1714806639	Y-box factor homolog [Manduca sexta]	Manduca	2	14	1	0.16
768445304	aldehyde dehydrogenase X, mitochondrial like [Plutella xylostella]	Plutella	6	68	1	0.16
1714840381	proteasome subunit beta type-1 [Manduca sexta]	Manduca	3	14	3	0.16
1714826196	serine-protein kinase ATM [Manduca sexta]	Manduca	1	3	1	0.15
1714807269	lysozyme-like [Manduca sexta]	Manduca	1	1	1	0.14
1714835304	protein lethal(2)essential for life-like [Manduca sexta]	Manduca	3	27	1	0.13
1714852506	protein ROP-like [Manduca sexta]	Manduca	5	21	1	0.13
1714826959	poly(U)-binding-splicing factor half pint [Manduca sexta]	Manduca	1	1	1	0.13
1714838135	sorting nexin lst-4 [Manduca sexta]	Manduca	2	7	2	0.13
768435479	digestive cysteine proteinase 2 isoform X1 [Plutella xylostella]	Plutella	3	16	1	0.13
1714850655	pyruvate carboxylase, mitochondrial-like [Manduca sexta]	Manduca	7	43	1	0.12
1714818818	uncharacterized protein LOC115445497 [Manduca sexta]	Manduca	1	2	1	0.11
768420790	gelsolin, partial [Plutella xylostella]	Manduca and Plutella	3	7	3	0.10
768436449	uncharacterized protein LOC105390160 [Plutella xylostella]	Plutella	4	16	4	0.10
768409687	titin [Plutella xylostella]	Plutella	5	15	5	0.09
768435469	coactosin-like protein isoform X1 [Plutella xylostella]	Manduca and Plutella	2	12	2	0.09

1174098354	basic juvenile hormone-suppressible protein 2-like precursor [Plutella xylostella]	Plutella	5	19	4	0.09
1714794490	LOW QUALITY PROTEIN: ubiquilin-1-like [Manduca sexta]	Manduca	6	27	1	0.07
1714820997	thymosin beta isoform X1 [Manduca sexta]	Manduca	4	20	4	0.06
822092754	uncharacterized protein LOC105388666 [Plutella xylostella]	Plutella	18	131	6	0.05
1714835288	heat shock protein 68-like [Manduca sexta]	Manduca	18	129	2	0.05
1714789068	uncharacterized protein LOC115455834 [Manduca sexta]	Manduca	5	31	4	0.04
1714794739	heat shock protein 68-like [Manduca sexta]	Manduca	5	42	2	0.04
1714790048	protein lethal(2)essential for life-like [Manduca sexta]	Manduca	3	9	3	0.04
1714782947	uncharacterized protein LOC115444227 [Manduca sexta]	Manduca	8	20	5	0.03
768417229	heat shock protein 68-like [Plutella xylostella]	Plutella	14	131	1	0.03
1714785079	protein lethal(2)essential for life-like [Manduca sexta]	Manduca	5	35	3	0.02
607029573	small heat shock protein [Plutella xylostella]	Plutella	3	24	2	0.01

Supplementary data file 2. 2-fold change (FC) difference and <0.05 FDR adjusted p-value significance; the 455 proteins were identified as dysregulated from *P. lilacinum* infected larvae when compared with the control sample.

Accession no.	Protein	Organism	# Pept		# Unique Peptides	Fold-change difference
768443294	reticulon-1-A isoform X1 [Plutella xylostella]	Plutella	2	12	1	71.80
1714791788	LOW QUALITY PROTEIN: ryanodine receptor [Manduca sexta]	Manduca	11	26	5	25.03
1714785471	AMP deaminase 2 isoform X1 [Manduca sexta]	Manduca	6	7	1	17.47
768431272	calcium uniporter protein, mitochondrial like isoform X1 [Plutella xylostella]	Manduca and Plutella	1	10	1	16.47

1714843099	neurochondrin homolog [Manduca sexta]	Manduca	5	17	1	13.27
1194641147	phosphate carrier 1 [Plutella xylostella]	Plutella	6	62	1	10.69
768417308	sodium/potassium-transporting ATPase subunit beta-2 [Plutella xylostella]	Manduca and Plutella	2	18	2	9.31
1714784318	ATP synthase lipid-binding protein, mitochondrial [Manduca sexta]	Manduca	1	39	1	8.76
768439318	V-type proton ATPase 116 kDa subunit a isoform 1 isoform X3 [Plutella xylostella]	Plutella	12	60	2	8.13
768415447	calcium-transporting ATPase sarcoplasmic/endoplasmic reticulum type isoform X1 [Plutella xylostella]	Plutella	21	229	2	7.81
1714824983	synaptic vesicle glycoprotein 2C-like isoform X1 [Manduca sexta]	Manduca	1	6	1	7.70
1714843767	protein transport protein Sec61 subunit alpha [Manduca sexta]	Manduca	9	68	9	7.44
1714842681	putative pre-mRNA-splicing factor ATP dependent RNA helicase PRP1 [Manduca sexta]	Manduca	6	18	6	7.34
768410384	translational activator GCN1-like [Plutella xylostella]	Plutella	5	16	5	7.29
768431223	LOW QUALITY PROTEIN: interleukin enhancer-binding factor 2 homolog [Plutella xylostella]	Plutella	3	10	2	7.12
1714837734	innexin inx2 [Manduca sexta]	Manduca	2	8	2	6.99
768414774	V-type proton ATPase subunit H isoform X1 [Plutella xylostella]	Plutella	16	92	2	6.43
1714829531	dolichyl-diphosphooligosaccharide--protein glycosyltransferase subunit STT3A [Manduca sexta]	Manduca	4	13	4	6.23
768413506	neurochondrin homolog [Plutella xylostella]	Plutella	5	23	1	6.01
1714820720	vacuolar protein sorting-associated protein 35 isoform X1 [Manduca sexta]	Manduca	2	9	2	5.79
1127252922	UDP-glycosyltransferase-42F1 [Plutella xylostella]	Plutella	1	3	1	5.75
1714843905	lysine--tRNA ligase isoform X1 [Manduca sexta]	Manduca	5	40	2	5.59
768429978	programmed cell death protein 6 isoform X1 [Plutella xylostella]	Plutella	3	13	3	5.47
165932398	cytochrome c oxidase subunit II (mitochondrion) [Manduca sexta]	Manduca	5	35	1	5.46

1714839839	UDP-glucuronosyltransferase 2B4-like [Manduca sexta]	Manduca	2	10	1	5.38
1714847100	UDP-glucuronosyltransferase 2B4-like isoform X1 [Manduca sexta]	Manduca	3	12	2	5.35
768444918	clustered mitochondria protein homolog [Plutella xylostella]	Plutella	7	19	2	5.32
768408066	hexokinase type 2 isoform X1 [Plutella xylostella]	Plutella	10	32	2	5.30
1714825426	importin-5 [Manduca sexta]	Manduca	9	51	9	5.28
1714818289	synaptojanin-1 [Manduca sexta]	Manduca	1	6	1	5.17
768442606	vesicle-trafficking protein SEC22b [Plutella xylostella]	Plutella	4	9	4	5.11
768421373	glutamate synthase [NADH], amyloplastic like [Plutella xylostella]	Plutella	7	20	3	5.05
768442023	ATP-dependent RNA helicase WM6 [Plutella xylostella]	Plutella	12	53	1	4.85

**Accession no.**	**Protein**	**Organism**	**# Pept**		**# Unique Peptides**	**Fold-change difference**
1714817478	translocon-associated protein subunit gamma [Manduca sexta]	Manduca	2	19	2	4.85
1714787824	Krueppel homolog 2 [Manduca sexta]	Manduca	1	7	1	4.76
1714802768	V-type proton ATPase 16 kDa proteolipid subunit [Manduca sexta]	Manduca	2	36	2	4.76
768418561	vacuolar protein sorting-associated protein 29 [Plutella xylostella]	Plutella	2	10	2	4.74
1714854095	phosphate carrier protein, mitochondrial like [Manduca sexta]	Manduca	6	53	1	4.49
768416141	zinc finger protein 816-like [Plutella xylostella]	Plutella	1	4	1	4.36
1714838480	metal transporter CNNM4-like [Manduca sexta]	Manduca	5	27	5	4.29
768416529	tryptophan 2,3-dioxygenase-like [Plutella xylostella]	Plutella	3	15	3	4.26
1714824089	piwi-like protein Siwi [Manduca sexta]	Manduca	7	24	5	4.12
1714833646	synaptotagmin 1 isoform X1 [Manduca sexta]	Manduca	2	5	2	3.99
768422560	pre-mRNA-processing-splicing factor 8 [Plutella xylostella]	Manduca and Plutella	6	12	6	3.97

1714854061	nicotinate phosphoribosyltransferase isoform X1 [Manduca sexta]	Manduca	1	9	1	3.92
1714821278	thioredoxin reductase-like selenoprotein T homolog CG3887 [Manduca sexta]	Manduca	2	17	2	3.92
1714835193	splicing factor 3B subunit 1 [Manduca sexta]	Manduca	3	5	3	3.92
768448059	coatomer subunit beta'-like [Plutella xylostella]	Plutella	5	17	3	3.91
768419379	muscle M-line assembly protein unc-89- like [Plutella xylostella]	Plutella	9	42	4	3.90
1714790939	coatomer subunit gamma [Manduca sexta]	Manduca	6	9	4	3.87
768415371	sodium/potassium-transporting ATPase subunit alpha isoform X1 [Plutella xylostella]	Plutella	29	148	29	3.85
768442347	probable N-acetyltransferase san [Plutella xylostella]	Plutella	2	9	1	3.83
1714858387	ADP,ATP carrier protein [Manduca sexta]	Manduca	16	204	2	3.82
488444153	serine/threonine-protein phosphatase 2A catalytic subunit [Plutella xylostella]	Manduca and Plutella	5	26	5	3.78
1714824281	1,4-alpha-glucan-branching enzyme [Manduca sexta]	Manduca	5	22	2	3.77
1714806575	eukaryotic translation initiation factor 4 gamma 3-like isoform X1 [Manduca sexta]	Manduca	3	10	3	3.76
1714845569	eukaryotic translation initiation factor 3 subunit K [Manduca sexta]	Manduca	7	26	6	3.73
768422071	plastin-3 isoform X3 [Plutella xylostella]	Manduca and Plutella	8	38	7	3.71
768443734	phosphoribosylformylglycinamidine synthase [Plutella xylostella]	Plutella	7	64	3	3.70
1714835219	serine/threonine-protein phosphatase 2B catalytic subunit 3-like [Manduca sexta]	Manduca	10	25	10	3.67
1714819420	D-beta-hydroxybutyrate dehydrogenase, mitochondrial [Manduca sexta]	Manduca	6	46	2	3.64
1714836738	V-type proton ATPase subunit d [Manduca sexta]	Manduca	5	18	2	3.60
768449720	coatomer subunit beta-like [Plutella xylostella]	Plutella	5	16	4	3.59
1714858948	dolichyl-diphosphooligosaccharide-- protein glycosyltransferase	Manduca	6	48	6	3.59

	subunit 1, partial [Manduca sexta]					
768424392	probable citrate synthase 2, mitochondrial [Plutella xylostella]	Plutella	7	57	4	3.55

Accession no.	Protein	Organism	# Pept		# Unique Peptides	Fold-change difference
1714836771	aromatic-L-amino-acid decarboxylase [Manduca sexta]	Manduca	2	12	1	3.54
768421871	cell differentiation protein RCD1 homolog [Plutella xylostella]	Plutella	1	1	1	3.54
1714815611	dipeptidyl peptidase 3 isoform X1 [Manduca sexta]	Manduca	3	25	1	3.51
768445010	clathrin heavy chain isoform X1 [Plutella xylostella]	Plutella	33	177	3	3.46
1714796863	calcium-binding mitochondrial carrier protein Aralar1 isoform X1 [Manduca sexta]	Manduca	7	40	5	3.44
1714833962	aspartate aminotransferase, cytoplasmic [Manduca sexta]	Manduca	3	33	3	3.43
1714794110	puromycin-sensitive aminopeptidase [Manduca sexta]	Manduca	10	40	6	3.42
787071490	adenine nucleotide translocase insect3 [Plutella xylostella]	Plutella	16	203	2	3.37
768428328	puromycin-sensitive aminopeptidase isoform X1 [Plutella xylostella]	Plutella	6	30	2	3.34
768430159	arginine--tRNA ligase, cytoplasmic [Plutella xylostella]	Plutella	4	20	1	3.33
768441273	scavenger receptor class B member 1 isoform X1 [Plutella xylostella]	Manduca and Plutella	2	7	2	3.31
1714787675	amino acid transporter AVT1B-like [Manduca sexta]	Manduca	1	7	1	3.30
768407834	transmembrane emp24 domain-containing protein bai [Plutella xylostella]	Plutella	4	36	4	3.29
1714812709	coatomer subunit alpha isoform X2 [Manduca sexta]	Manduca	14	28	3	3.23
768407893	presequence protease, mitochondrial-like [Plutella xylostella]	Plutella	3	9	2	3.15
768436859	kynurenine 3-monooxygenase-like	Plutella	2	6	2	3.14

	[Plutella xylostella]					
768427005	uncharacterized protein LOC105385824 [Plutella xylostella]	Plutella	5	21	2	3.10
1714816610	glutamine--fructose-6-phosphate aminotransferase [isomerizing] 1-like [Manduca sexta]	Manduca	4	14	4	3.08
1714845899	proline dehydrogenase 1, mitochondrial like [Manduca sexta]	Manduca	5	44	5	3.07
1714797181	40S ribosomal protein SA [Manduca sexta]	Manduca	10	118	2	3.07
1714779221	N-alpha-acetyltransferase 15, NatA auxiliary subunit [Manduca sexta]	Manduca	4	11	4	3.07
1714779918	casein kinase II subunit alpha isoform X1 [Manduca sexta]	Manduca	8	17	8	3.07
768446211	ATP-dependent RNA helicase Ddx1-like [Plutella xylostella]	Plutella	2	7	2	3.06
768428731	uncharacterized protein LOC105386610 [Plutella xylostella]	Manduca and Plutella	1	10	1	3.04
768409771	V-type proton ATPase 116 kDa subunit a isoform 1-like isoform X1 [Plutella xylostella]	Plutella	8	56	8	3.03
1714789770	AFG3-like protein 2 [Manduca sexta]	Manduca	13	41	3	3.02
1714780065	translocating chain-associated membrane protein 1 [Manduca sexta]	Manduca	1	9	1	3.00
1714798516	uncharacterized protein LOC115441016 [Manduca sexta]	Manduca	13	96	13	2.97
768440535	laminin subunit alpha-like, partial [Plutella xylostella]	Plutella	6	30	2	2.96
1714827818	myosin heavy chain, muscle isoform X14 [Manduca sexta]	#N/A	169	2916	0	2.95
1714816500	26S proteasome non-ATPase regulatory subunit 6 [Manduca sexta]	Manduca	5	27	3	2.94
1714830307	threonine--tRNA ligase, cytoplasmic isoform X1 [Manduca sexta]	Manduca	11	52	11	2.92

**Accession no.**	**Protein**	**Organism**	**# Pept**		**# Unique Peptides**	**Fold-change difference**
1714797205	ubiquitin carboxyl-terminal hydrolase 7- like isoform X1 [Manduca sexta]	Manduca	11	50	7	2.92

1714790982	NADH-ubiquinone oxidoreductase 49 kDa subunit [Manduca sexta]	Manduca	8	45	8	2.92
1714784806	LOW QUALITY PROTEIN: tripeptidyl peptidase 2 [Manduca sexta]	Manduca	2	13	2	2.91
1714844215	tubulin beta chain-like [Manduca sexta]	Manduca	14	118	3	2.91
768438622	calcium/calmodulin-dependent protein kinase type II alpha chain [Plutella xylostella]	Plutella	5	26	1	2.91
1714795867	xanthine dehydrogenase [Manduca sexta]	Manduca	4	20	4	2.90
1714851726	protein krasavietz [Manduca sexta]	Manduca	5	14	5	2.89
1714786476	26S proteasome non-ATPase regulatory subunit 12 [Manduca sexta]	Manduca	7	38	7	2.88
768426480	serine/threonine-protein phosphatase PP2A 65 kDa regulatory subunit [Plutella xylostella]	Plutella	11	64	1	2.88
1714808308	clathrin heavy chain [Manduca sexta]	Manduca	40	202	10	2.86
768433865	eukaryotic translation initiation factor 3 subunit B [Plutella xylostella]	Plutella	12	46	12	2.85
1714804170	calcium-transporting ATPase sarcoplasmic/endoplasmic reticulum type isoform X1 [Manduca sexta]	Manduca	26	266	7	2.82
1714812923	NADP-dependent malic enzyme-like isoform X1 [Manduca sexta]	Manduca	10	58	10	2.81
1714830135	probable aminopeptidase NPEPL1 isoform X1 [Manduca sexta]	Manduca	2	11	2	2.79
1714798431	uncharacterized protein LOC115440997 isoform X1 [Manduca sexta]	Manduca	11	102	3	2.76
768429249	LOW QUALITY PROTEIN: 26S proteasome non-ATPase regulatory subunit 1-like [Plutella xylostella]	Plutella	12	64	2	2.72
768439997	lysine--tRNA ligase isoform X1 [Plutella xylostella]	Plutella	5	46	2	2.71
1714805039	dolichyl-diphosphooligosaccharide--protein glycosyltransferase 48 kDa subunit [Manduca sexta]	Manduca	5	30	5	2.70
1714844507	endocuticle structural glycoprotein SgAbd 8-like [Manduca sexta]	Manduca	3	7	3	2.67
768433761	uncharacterized protein C05D11.1-like [Plutella xylostella]	Plutella	2	9	2	2.66
1714834105	juvenile hormone epoxide hydrolase-like [Manduca sexta]	Manduca	1	8	1	2.63

768429473	D-beta-hydroxybutyrate dehydrogenase, mitochondrial isoform X1 [Plutella xylostella]	Plutella	5	27	1	2.62
1714841381	ADP-ribosylation factor-like protein 8 [Manduca sexta]	Manduca	3	7	3	2.61
1714816572	glutamate--cysteine ligase catalytic subunit [Manduca sexta]	Manduca	2	10	2	2.59
1714836753	trifunctional enzyme subunit alpha, mitochondrial [Manduca sexta]	Manduca	5	43	1	2.58
1714832549	LOW QUALITY PROTEIN: multidrug resistance protein homolog 49-like [Manduca sexta]	Manduca	3	8	3	2.57
1714839511	putative tricarboxylate transport protein, mitochondrial [Manduca sexta]	Manduca	5	32	5	2.57
768448701	uncharacterized protein LOC105396107 [Plutella xylostella]	Plutella	1	1	1	2.55
1714817988	cytochrome c oxidase subunit 4 isoform 1, mitochondrial-like [Manduca sexta]	Manduca	2	12	2	2.54
1714823704	dolichyl-diphosphooligosaccharide-- protein glycosyltransferase subunit 2 [Manduca sexta]	Manduca	2	10	2	2.54
768411720	2-oxoglutarate dehydrogenase, mitochondrial [Plutella xylostella]	Plutella	28	289	1	2.53

**Accession no.**	**Protein**	**Organism**	**# Pept**		**# Unique Peptides**	**Fold-change difference**
1714795408	cAMP-dependent protein kinase catalytic subunit 1 [Manduca sexta]	Manduca	7	19	7	2.50
768443470	purine nucleoside phosphorylase-like isoform X1 [Plutella xylostella]	Manduca and Plutella	1	10	1	2.47
768424601	ATP-binding cassette sub-family G member 1-like [Plutella xylostella]	Manduca and Plutella	1	9	1	2.47
1714833749	glycogen phosphorylase [Manduca sexta]	Manduca	23	162	14	2.47
1714798768	phosphoribosylformylglycinamidine synthase [Manduca sexta]	Manduca	10	103	6	2.45
1714816107	myrosinase 1-like [Manduca sexta]	Manduca	1	5	1	2.44
1714841515	reticulon-1 isoform X1 [Manduca sexta]	Manduca	2	9	1	2.44
1714836369	ATP-citrate synthase [Manduca sexta]	Manduca	39	325	14	2.43

1714795342	mitochondrial proton/calcium exchanger protein [Manduca sexta]	Manduca	7	33	7	2.42
768421658	guanine nucleotide-binding protein G(o) subunit alpha isoform X1 [Plutella xylostella]	Manduca and Plutella	8	34	8	2.42
1714828155	ras-related protein Ral-a isoform X1 [Manduca sexta]	Manduca	1	1	1	2.40
768441490	cytosolic purine 5'-nucleotidase isoform X1 [Plutella xylostella]	Plutella	4	43	4	2.39
1714837777	116 kDa U5 small nuclear ribonucleoprotein component [Manduca sexta]	Manduca	5	10	5	2.38
768446145	FUN14 domain-containing protein 1-like isoform X1 [Plutella xylostella]	Manduca and Plutella	1	4	1	2.37
768439586	calcium-binding mitochondrial carrier protein Aralar1-like [Plutella xylostella]	Plutella	3	23	1	2.36
768432945	extended synaptotagmin-2-A-like, partial [Plutella xylostella]	Plutella	8	27	2	2.34
768413292	mitochondrial-processing peptidase subunit alpha [Plutella xylostella]	Plutella	2	7	1	2.34
1714785566	atlastin isoform X1 [Manduca sexta]	Manduca	2	4	2	2.32
1714786519	alpha-actinin, sarcomeric isoform X1 [Manduca sexta]	Manduca	61	664	16	2.32
1714796960	tolloid-like protein 1 [Manduca sexta]	Manduca	1	8	1	2.31
768408615	alpha-actinin, sarcomeric-like [Plutella xylostella]	Plutella	50	555	5	2.29
1714789907	plasma membrane calcium-transporting ATPase 2 isoform X1 [Manduca sexta]	Manduca	25	139	25	2.29
1714788345	ATP-dependent RNA helicase WM6 [Manduca sexta]	Manduca	18	88	7	2.29
987437870	60 kDa heat shock protein, mitochondrial like [Plutella xylostella]	Plutella	18	243	15	2.27
768426118	regulator of nonsense transcripts 1-like [Plutella xylostella]	Manduca and Plutella	5	13	5	2.27
768425216	eukaryotic translation initiation factor 3 subunit K [Plutella xylostella]	Plutella	4	9	3	2.27
768414045	S-formylglutathione hydrolase [Plutella xylostella]	Plutella	3	25	3	2.26
768419808	NADP-dependent malic enzyme isoform X1 [Plutella xylostella]	Plutella	8	43	3	2.25
1714836224	ATP synthase subunit gamma,	Manduca	3	40	2	2.25

	mitochondrial [Manduca sexta]					
671871149	P450 CYP6 family protein 6 [Plutella xylostella]	Plutella	1	11	1	2.23
768433478	myosin heavy chain, muscle isoform X15 [Plutella xylostella]	Plutella	158	2787	2	2.22
1714807772	integrin beta-PS [Manduca sexta]	Manduca	2	6	2	2.22
768410192	UDP-glucose:glycoprotein glucosyltransferase [Plutella xylostella]	Plutella	3	14	1	2.22
768443210	tripeptidyl-peptidase 2 [Plutella xylostella]	Plutella	2	6	2	2.21
1714782743	long-chain-fatty-acid--CoA ligase 4 isoform X1 [Manduca sexta]	Manduca	4	40	2	2.21
768423690	bifunctional glutamate/proline--tRNA ligase isoform X1 [Plutella xylostella]	Plutella	7	34	6	2.19

**Accession no.**	**Protein**	**Organism**	**# Pept**		**# Unique Peptides**	**Fold-change difference**
1714853725	L-asparaginase-like [Manduca sexta]	Manduca	3	8	3	2.19
768451820	transaldolase-like [Plutella xylostella]	Plutella	3	13	2	2.19
1714786893	protein arginine N-methyltransferase 1 [Manduca sexta]	Manduca	4	21	4	2.15
768426977	carnitine O-palmitoyltransferase 2, mitochondrial-like [Plutella xylostella]	Plutella	1	2	1	2.15
1127252882	Fatty acid synthase-4, partial [Plutella xylostella]	Plutella	3	35	1	2.14
768443446	uncharacterized protein K02A2.6-like [Plutella xylostella]	Plutella	1	1	1	2.13
1714818345	alpha-aminoadipic semialdehyde synthase, mitochondrial [Manduca sexta]	Manduca	12	80	3	2.13
1714817409	26S proteasome non-ATPase regulatory subunit 2 [Manduca sexta]	Manduca	14	98	4	2.13
1714823275	putative ATP synthase subunit f, mitochondrial [Manduca sexta]	Manduca	1	18	1	2.13
164683438	eukaryotic initiation factor 4A [Plutella xylostella]	Plutella	19	175	3	2.13
1714814714	uncharacterized protein LOC115444704, partial [Manduca sexta]	Manduca	51	186	34	2.12

768424454	V-type proton ATPase subunit C isoform X1 [Plutella xylostella]	Plutella	11	77	3	2.12
671871185	P450 CYP6 family protein 10 [Plutella xylostella]	Plutella	2	10	2	2.11
768438040	ATP-binding cassette sub-family E member 1 [Plutella xylostella]	Plutella	8	44	8	2.10
768443222	proteasome activator complex subunit 3 isoform X1 [Plutella xylostella]	Plutella	4	13	4	2.10
117970173	pxS-adenosyl-L-homocysteine hydrolase [Plutella xylostella]	Plutella	13	137	2	2.10
768433673	C-terminal-binding protein [Plutella xylostella]	Plutella	7	26	7	2.09
1714847252	serine/threonine-protein phosphatase 2A 65 kDa regulatory subunit A alpha isoform [Manduca sexta]	Manduca	14	82	4	2.09
1714822723	acetyl-CoA carboxylase isoform X1 [Manduca sexta]	Manduca	34	186	19	2.09
768445239	T-complex protein 1 subunit alpha [Plutella xylostella]	Plutella	13	101	1	2.09
1714837691	mitochondrial import receptor subunit TOM40 homolog 1-like [Manduca sexta]	Manduca	4	38	4	2.08
1714813192	ATP synthase subunit b, mitochondrial [Manduca sexta]	Manduca	1	14	1	2.08
768410047	uncharacterized protein LOC105387140 isoform X1 [Plutella xylostella]	Plutella	10	100	2	2.07
1127252884	Fatty acid synthase-5 [Plutella xylostella]	Plutella	2	11	1	2.07
768418331	glyoxylate reductase/hydroxypyruvate reductase-like [Plutella xylostella]	Plutella	1	4	1	2.07
1714806071	dihydropyrimidine dehydrogenase [NADP(+)] [Manduca sexta]	Manduca	11	46	11	2.07
1714781838	venom carboxylesterase-6-like [Manduca sexta]	Manduca	1	6	1	2.06
768433466	myosin heavy chain, muscle isoform X9 [Plutella xylostella]	Plutella	160	2817	1	2.06
768438558	glucose-6-phosphate isomerase [Plutella xylostella]	Plutella	6	68	2	2.06
768419683	adipocyte plasma membrane-associated protein isoform X1 [Plutella xylostella]	Plutella	1	7	1	2.05
768425765	T-complex protein 1 subunit	Plutella	10	56	7	2.05

	beta-like [Plutella xylostella]					
768450471	uncharacterized protein LOC105397069 [Plutella xylostella]	Plutella	1	9	1	2.04
768430234	importin subunit beta-1-like [Plutella xylostella]	Plutella	2	15	2	2.04

**Accession no.**	**Protein**	**Organism**	**# Pept**		**# Unique Peptides**	**Fold-change difference**
1714848642	pentatricopeptide repeat-containing protein 2, mitochondrial-like [Manduca sexta]	Manduca	1	8	1	2.02
1714851519	V-type proton ATPase 116 kDa subunit a isoform X3 [Manduca sexta]	Manduca	13	63	3	2.02
768424460	ATP synthase subunit gamma, mitochondrial [Plutella xylostella]	Plutella	4	29	3	2.02
1714815555	ruvB-like helicase 1 [Manduca sexta]	Manduca	6	29	6	2.01
1714856098	H/ACA ribonucleoprotein complex subunit 4-like [Manduca sexta]	Manduca	10	63	10	2.01
1714843560	cytochrome P450 9e2-like [Manduca sexta]	Manduca	3	19	3	2.00
1714799935	adenylate kinase [Manduca sexta]	Manduca	8	64	6	0.50
1714821271	COP9 signalosome complex subunit 9 [Manduca sexta]	Manduca	1	6	1	0.50
768433032	CCHC-type zinc finger protein CG3800 [Plutella xylostella]	Plutella	2	19	2	0.50
1714812765	probable small nuclear ribonucleoprotein Sm D2 [Manduca sexta]	Manduca	7	51	7	0.50
1714819213	heterogeneous nuclear ribonucleoprotein A1-like [Manduca sexta]	Manduca	3	23	3	0.50
1714815507	PDZ and LIM domain protein Zasp isoform X6 [Manduca sexta]	Manduca	11	94	8	0.49
1714832101	12 kDa FK506-binding protein-like [Manduca sexta]	Manduca	3	16	3	0.49
1714843374	tropomyosin-2 isoform X13 [Manduca sexta]	Manduca	39	739	8	0.49
1714780041	probable elongation factor 1-delta isoform X1 [Manduca sexta]	Manduca	1	13	1	0.49
768437513	protein held out wings [Plutella xylostella]	Plutella	3	6	3	0.49

768443164	flavin reductase (NADPH) [Plutella xylostella]	Plutella	1	12	1	0.49
1714838106	uncharacterized protein LOC115449737 [Manduca sexta]	Manduca	1	9	1	0.49
1714814099	40S ribosomal protein S20 [Manduca sexta]	Manduca	3	26	3	0.49
770075562	cytochrome c [Plutella xylostella]	Plutella	4	46	4	0.49
1714852094	uncharacterized protein LOC115453316 [Manduca sexta]	Manduca	1	16	1	0.49
1714824563	alpha-catulin isoform X1 [Manduca sexta]	Manduca	1	9	1	0.49
1714808844	ribosome-binding protein 1 isoform X1 [Manduca sexta]	Manduca	3	11	3	0.49
1714852366	uncharacterized protein LOC115455253 [Manduca sexta]	Manduca	1	9	1	0.48
768447608	bifunctional 3'-phosphoadenosine 5'-phosphosulfate synthase [Plutella xylostella]	Plutella	8	42	4	0.48
1714800027	chromobox protein homolog 1-like isoform X1 [Manduca sexta]	Manduca	3	12	3	0.48
1714794276	heterogeneous nuclear ribonucleoprotein M isoform X1 [Manduca sexta]	Manduca	6	39	6	0.48
1714837793	endoplasmic reticulum chaperone BiP isoform X2 [Manduca sexta]	Manduca	32	494	4	0.48
768447669	phosphoglycerate mutase 1-like [Plutella xylostella]	Plutella	7	81	7	0.48
1714828204	titin-like isoform X1 [Manduca sexta]	Manduca	2	5	2	0.48
768425048	heterogeneous nuclear ribonucleoprotein 87F-like [Plutella xylostella]	Plutella	7	66	5	0.48
1714846630	aldo-keto reductase family 1 member B1- like [Manduca sexta]	Manduca	5	51	3	0.47
1714787004	eukaryotic translation initiation factor 1A, X-chromosomal [Manduca sexta]	Manduca	5	13	5	0.47

**Accession no.**	**Protein**	**Organism**	**# Pept**		**# Unique Peptides**	**Fold-change difference**
1714817426	enolase [Manduca sexta]	Manduca	11	210	11	0.47
49532856	Ribosomal protein L36A [Plutella xylostella]	Plutella	6	73	6	0.47

1714782021	26S proteasome regulatory subunit 6B [Manduca sexta]	Manduca	21	133	21	0.47
768436047	aldose reductase-like [Plutella xylostella]	Plutella	2	16	1	0.47
1714800745	protein disulfide-isomerase A3 [Manduca sexta]	Manduca	3	32	2	0.47
768423908	uncharacterized protein LOC105384358 isoform X1 [Plutella xylostella]	Plutella	8	88	1	0.47
768430181	putative RNA-binding protein 15 [Plutella xylostella]	Plutella	4	14	4	0.47
1714804470	ubiquitin-fold modifier 1 isoform X2 [Manduca sexta]	Manduca	4	26	4	0.47
1714842438	ubiquitin-conjugating enzyme E2 G1 isoform X1 [Manduca sexta]	Manduca	2	8	2	0.46
768412058	cytochrome b-c1 complex subunit 7-like [Plutella xylostella]	Plutella	1	9	1	0.46
1714830944	UV excision repair protein RAD23 homolog A [Manduca sexta]	Manduca	5	20	5	0.46
1714843403	tropomyosin-1 [Manduca sexta]	Manduca	29	672	27	0.46
1714819169	heterogeneous nuclear ribonucleoprotein 87F-like [Manduca sexta]	Manduca	4	35	2	0.46
1714833115	aldo-keto reductase AKR2E4-like isoform X1 [Manduca sexta]	Manduca	2	10	2	0.46
768420291	neprilysin-11 isoform X1 [Plutella xylostella]	Plutella	2	5	2	0.46
1714808547	LOW QUALITY PROTEIN: serpin A9-like [Manduca sexta]	Manduca	1	22	1	0.46
1714820847	KH domain-containing, RNA-binding, signal transduction-associated protein 2-like [Manduca sexta]	Manduca	2	14	2	0.46
1714810293	heat shock factor-binding protein 1 isoform X1 [Manduca sexta]	Manduca	6	14	6	0.46
1714786330	L-xylulose reductase-like [Manduca sexta]	Manduca	5	39	4	0.46
1714793625	ubiquitin domain-containing protein UBFD1-like [Manduca sexta]	Manduca	1	2	1	0.45
768415639	triosephosphate isomerase [Plutella xylostella]	Plutella	6	81	2	0.45
770075529	60S ribosomal protein L31 [Plutella xylostella]	Plutella	6	86	2	0.45
1714823019	enhancer of rudimentary homolog [Manduca sexta]	Manduca	3	14	3	0.45

1714844700	aldo-keto reductase family 1 member B1- like isoform X1 [Manduca sexta]	Manduca	6	72	5	0.45
768447564	26S protease regulatory subunit 6A-B [Plutella xylostella]	Plutella	20	144	2	0.45
1714820445	branched-chain-amino-acid aminotransferase, cytosolic [Manduca sexta]	Manduca	2	5	2	0.45
1714811260	aldehyde dehydrogenase X, mitochondrial like [Manduca sexta]	Manduca	7	62	2	0.45
1714814635	nuclear migration protein nudC [Manduca sexta]	Manduca	5	29	5	0.45
768412076	lamin Dm0-like [Plutella xylostella]	Plutella	21	175	5	0.45
768413904	heat shock 70 kDa protein cognate 3-like [Plutella xylostella]	Plutella	33	480	5	0.45
768408976	protein D2-like isoform X1 [Plutella xylostella]	Plutella	4	27	4	0.44
1714801062	rho GDP-dissociation inhibitor 1 isoform X2 [Manduca sexta]	Manduca	5	33	5	0.44
1714814022	tubulin beta chain-like isoform X2 [Manduca sexta]	Manduca	19	264	7	0.44
1714850531	clathrin light chain-like isoform X1 [Manduca sexta]	Manduca	2	16	2	0.44

**Accession no.**	**Protein**	**Organism**	**# Pept**		**# Unique Peptides**	**Fold-change difference**
1714850878	endothelial differentiation-related factor 1 homolog isoform X1 [Manduca sexta]	Manduca	2	14	2	0.44
1714816672	extended synaptotagmin-2-A isoform X1 [Manduca sexta]	Manduca	9	35	3	0.44
768428604	thioredoxin-like protein 1 [Plutella xylostella]	Plutella	2	22	2	0.44
1714829845	SUMO-conjugating enzyme UBC9-A [Manduca sexta]	Manduca and Plutella	8	32	8	0.44
768421686	protein lethal(2)essential for life-like [Plutella xylostella]	Plutella	3	34	3	0.44
1714856382	NEDD8 [Manduca sexta]	Manduca	3	29	3	0.44
768447461	myosin regulatory light chain 2-like [Plutella xylostella]	Plutella	4	108	4	0.43
1714818754	membrane-bound alkaline phosphatase like [Manduca sexta]	Manduca	1	6	1	0.43

1714819196	prefoldin subunit 6 [Manduca sexta]	Manduca	5	32	5	0.43
768419209	stress-induced-phosphoprotein 1-like [Plutella xylostella]	Plutella	5	28	5	0.43
768420892	thioredoxin reductase 1, mitochondrial like isoform X1 [Plutella xylostella]	Plutella	3	20	1	0.43
1714803661	ras GTPase-activating protein-binding protein 2, partial [Manduca sexta]	Manduca	5	21	2	0.43
1714781802	calmodulin isoform X1 [Manduca sexta]	Manduca	14	159	13	0.43
1714780838	glycine-rich cell wall structural protein [Manduca sexta]	Manduca	2	32	2	0.43
1714812774	serine/threonine-protein phosphatase 2A 55 kDa regulatory subunit B alpha isoform isoform X1 [Manduca sexta]	Manduca	2	8	2	0.42
768411157	classical arabinogalactan protein 4-like [Plutella xylostella]	Plutella	1	8	1	0.42
1714785732	COP9 signalosome complex subunit 1 [Manduca sexta]	Manduca	3	4	3	0.42
1714815968	sialic acid synthase [Manduca sexta]	Manduca	4	21	2	0.42
1714788098	myosin regulatory light chain sqh [Manduca sexta]	Manduca	7	30	7	0.42
768449175	hydroxyacyl-coenzyme A dehydrogenase, mitochondrial-like [Plutella xylostella]	Plutella	5	44	1	0.42
768419247	proteasome subunit alpha type-6-like [Plutella xylostella]	Plutella	6	49	2	0.42
1714783673	uncharacterized protein LOC115445754 isoform X1 [Manduca sexta]	Manduca	1	10	1	0.42
1714843393	tropomyosin-1, isoforms 9A/A/B isoform X19 [Manduca sexta]	Manduca	22	310	6	0.41
1714793065	ubiquitin carboxyl-terminal hydrolase 5 [Manduca sexta]	Manduca	6	25	5	0.41
768408131	uncharacterized protein ZC395.10-like [Plutella xylostella]	Manduca and Plutella	1	5	1	0.41
768425018	glutaredoxin 3 [Plutella xylostella]	Plutella	2	23	2	0.41
1714800977	histone H3.3 [Manduca sexta]	Manduca and Plutella	5	73	1	0.41
1714843381	tropomyosin-2 isoform X15 [Manduca sexta]	#N/A	24	429	0	0.41
1714844337	proteasome subunit alpha type-6-like [Manduca sexta]	Manduca	7	52	3	0.41
1714822547	hepatocyte growth	Manduca	3	11	3	0.41

	factor-regulated tyrosine kinase substrate isoform X1 [Manduca sexta]					
1714784627	eukaryotic translation initiation factor 3 subunit A [Manduca sexta]	Manduca	1	10	1	0.41
1714800243	neurogenic locus notch homolog protein 1 [Manduca sexta]	Manduca	5	31	4	0.41
768447965	heat shock protein beta-1-like [Plutella xylostella]	Plutella	8	63	8	0.41
768423176	neurogenic locus notch homolog protein 4- like [Plutella xylostella]	Plutella	2	21	1	0.41
1714792933	LOW QUALITY PROTEIN: high mobility group protein D-like [Manduca sexta]	Manduca	1	33	1	0.40

**Accession no.**	**Protein**	**Organism**	**# Pept**		**# Unique Peptides**	**Fold-change difference**
1714846832	selenoprotein M-like [Manduca sexta]	Manduca	1	4	1	0.40
1714789564	U6 snRNA-associated Sm-like protein LSm6 [Manduca sexta]	Manduca	2	9	2	0.40
768420965	peptidyl-prolyl cis-trans isomerase B [Plutella xylostella]	Plutella	4	33	4	0.40
768427950	small nuclear ribonucleoprotein Sm D1 [Plutella xylostella]	Plutella	2	20	1	0.40
1714815438	peroxiredoxin-6 [Manduca sexta]	Manduca	2	8	2	0.40
768420532	tropomyosin-2 isoform X2 [Plutella xylostella]	Plutella	20	368	2	0.39
822092476	glyceraldehyde-3-phosphate dehydrogenase [Plutella xylostella]	Plutella	7	259	1	0.39
1714835189	vigilin isoform X2 [Manduca sexta]	Manduca	15	113	7	0.39
1714830170	triosephosphate isomerase [Manduca sexta]	Manduca	7	93	3	0.39
1714857497	bifunctional 3'-phosphoadenosine 5'-phosphosulfate synthase 2-like [Manduca sexta]	Manduca	5	32	1	0.39
1714799386	protein LSM14 homolog B isoform X1 [Manduca sexta]	Manduca	3	16	3	0.39
1714839079	ferritin subunit [Manduca sexta]	Manduca	1	9	1	0.39
1714790157	protein lethal(2)essential for life-like [Manduca sexta]	Manduca	6	93	4	0.39
768436581	arginine kinase isoform X1	Plutella	21	1095	1	0.39

	[Plutella xylostella]					
1714844694	alpha-centractin [Manduca sexta]	Manduca	5	13	5	0.38
1714805288	cytosolic carboxypeptidase 2-like [Manduca sexta]	Manduca	1	6	1	0.38
768445006	dehydrogenase/reductase SDR family member 4 [Plutella xylostella]	Plutella	1	9	1	0.38
1714815957	carbonic anhydrase 1 [Manduca sexta]	Manduca	1	6	1	0.38
768410779	serine/arginine repetitive matrix protein 1- like isoform X1 [Plutella xylostella]	Manduca and Plutella	2	10	2	0.38
1714786277	L-xylulose reductase-like [Manduca sexta]	Manduca	1	6	1	0.38
1714840539	calreticulin [Manduca sexta]	Manduca	8	92	4	0.38
1714851643	S-methyl-5'-thioadenosine phosphorylase like [Manduca sexta]	Manduca	2	7	2	0.38
1714851245	RNA transcription, translation and transport factor protein [Manduca sexta]	Manduca	2	3	2	0.38
768438732	U1 small nuclear ribonucleoprotein C [Plutella xylostella]	Manduca and Plutella	2	9	2	0.38
768415052	mesencephalic astrocyte-derived neurotrophic factor homolog [Plutella xylostella]	Manduca and Plutella	1	3	1	0.37
768445306	aldehyde dehydrogenase X, mitochondrial like [Plutella xylostella]	Plutella	9	77	3	0.37
1714795047	PC4 and SFRS1-interacting protein isoform X1 [Manduca sexta]	Manduca	1	6	1	0.37
1714795060	translation machinery-associated protein 7 homolog isoform X2 [Manduca sexta]	Manduca	3	9	3	0.37
768410802	LOW QUALITY PROTEIN: calumenin-A-like [Plutella xylostella]	Plutella	3	11	2	0.36
1714851484	histone H3-like [Manduca sexta]	Manduca	5	45	1	0.36
768410288	macrophage migration inhibitory factor like [Plutella xylostella]	Plutella	2	21	2	0.36
1714854753	phenoloxidase-activating factor 2-like, partial [Manduca sexta]	Manduca	2	18	2	0.36
768413216	U6 snRNA-associated Sm-like protein LSm3 [Plutella xylostella]	Plutella	1	8	1	0.36
768431908	myotrophin-like [Plutella xylostella]	Plutella	3	10	3	0.36
768425078	transmembrane protease serine 9-like [Plutella xylostella]	Manduca and Plutella	1	5	1	0.36

768447528	sorting nexin-2-like [Plutella xylostella]	Plutella	3	7	2	0.36
768448326	probable prefoldin subunit 4 [Plutella xylostella]	Plutella	1	10	1	0.36

**Accession no.**	**Protein**	**Organism**	**# Pept**		**# Unique Peptides**	**Fold-change difference**
1714851451	striatin isoform X1 [Manduca sexta]	Manduca	3	11	3	0.35
768428921	mRNA-decapping enzyme 1B-like [Plutella xylostella]	Manduca and Plutella	2	7	2	0.35
1714812652	pleckstrin homology domain-containing family F member 2 isoform X1 [Manduca sexta]	Manduca	2	4	2	0.35
1714830106	digestive cysteine proteinase 1 [Manduca sexta]	Manduca	3	14	1	0.35
1714847321	myosin-2 essential light chain isoform X1 [Manduca sexta]	Manduca	5	31	5	0.35
768445694	uncharacterized protein LOC105394551 [Plutella xylostella]	Plutella	2	8	2	0.35
1714799300	chitinase-like protein EN03 isoform X1	Manduca	4	186	4	0.35
1714802054	glutathione S-transferase 2-like isoform X1 [Manduca sexta]	Manduca	1	6	1	0.35
768443011	ubiquitin-fold modifier-conjugating enzyme 1 [Plutella xylostella]	Plutella	3	14	3	0.34
1714853512	26S proteasome regulatory subunit 4 [Manduca sexta]	Manduca	18	81	17	0.34
1714822245	uncharacterized protein LOC115446254 [Manduca sexta]	Manduca	3	13	3	0.34
1714813038	synapse-associated protein of 47 kDa isoform X1 [Manduca sexta]	Manduca	2	6	2	0.33
768414627	putative neuropeptide precursor protein isoform X1 [Plutella xylostella]	Plutella	4	10	4	0.33
1714796329	zinc-type alcohol dehydrogenase-like protein C1773.06c [Manduca sexta]	Manduca	1	4	1	0.33
1714819426	prefoldin subunit 3 [Manduca sexta]	Manduca	2	7	2	0.33
1025716978	thioredoxin-like protein [Plutella xylostella]	Plutella	2	100	2	0.33
1714802920	glycine N-methyltransferase isoform X1 [Manduca sexta]	Manduca	4	38	4	0.33

768418023	ATP-dependent RNA helicase dbp4-like [Plutella xylostella]	Manduca and Plutella	1	8	1	0.32
1714843350	tropomyosin-2 isoform X7 [Manduca sexta]	#N/A	29	533	0	0.32
1714832438	28S ribosomal protein S17, mitochondrial [Manduca sexta]	Manduca	2	6	2	0.32
768419735	troponin C, isoform 3-like [Plutella xylostella]	Plutella	9	118	4	0.32
1714834544	apolipophorin-3 [Manduca sexta]	Manduca	1	27	1	0.32
768412686	microtubule-associated protein RP/EB family member 3, partial [Plutella xylostella]	Plutella	3	27	2	0.31
49532918	cellular retinoic acid binding protein [Plutella xylostella]	Plutella	5	86	4	0.31
1714845982	general transcription factor IIF subunit 1- like [Manduca sexta]	Manduca	4	23	4	0.31
1714795275	transferrin [Manduca sexta]	Manduca	5	31	5	0.31
1714824465	uncharacterized protein LOC115446757 [Manduca sexta]	Manduca	1	5	1	0.30
1714818527	adenylyl cyclase-associated protein 1 isoform X1 [Manduca sexta]	Manduca	4	22	4	0.30
768415969	carbonic anhydrase 15-like [Plutella xylostella]	Plutella	1	3	1	0.30
768421038	vacuolar protein sorting-associated protein VTA1 homolog isoform X1 [Plutella xylostella]	Plutella	1	1	1	0.30
768440608	WW domain-binding protein 2 [Plutella xylostella]	Plutella	1	3	1	0.30
1714853717	U6 snRNA-associated Sm-like protein LSm4 [Manduca sexta]	Manduca	2	6	2	0.30
388252732	beta-1,3-glucan binding protein [Plutella xylostella]	Plutella	1	6	1	0.30
768418109	LDLR chaperone boca [Plutella xylostella]	Plutella	3	19	3	0.29
768431340	SPARC [Plutella xylostella]	Plutella	1	3	1	0.28

**Accession no.**	**Protein**	**Organism**	**# Pept**		**# Unique Peptides**	**Fold-change difference**
768451365	LOW QUALITY PROTEIN: alpha-2-macroglobulin receptor-associated protein like [Plutella xylostella]	Plutella	1	4	1	0.28

768431856	kinesin light chain-like [Plutella xylostella]	Plutella	3	7	2	0.28
1714788570	FK506-binding protein 2 [Manduca sexta]	Manduca	9	59	1	0.28
1714853488	signal recognition particle 54 kDa protein [Manduca sexta]	Manduca	5	25	5	0.28
1714825657	stromal cell-derived factor 2 [Manduca sexta]	Manduca	1	6	1	0.28
1714808375	alpha-tocopherol transfer protein-like [Manduca sexta]	Manduca	2	2	2	0.28
768431748	heterogeneous nuclear ribonucleoprotein K [Plutella xylostella]	Manduca and Plutella	2	10	2	0.28
768439126	transitional endoplasmic reticulum ATPase TER94 [Plutella xylostella]	Plutella	42	372	1	0.28
1714808268	sodium/calcium exchanger regulatory protein 1 [Manduca sexta]	Manduca	2	36	1	0.28
768445580	prefoldin subunit 2 [Plutella xylostella]	Plutella	1	8	1	0.27
768424642	chitooligosaccharidolytic beta-N acetylglucosaminidase [Plutella xylostella]	Plutella	1	2	1	0.27
768439252	troponin T, skeletal muscle isoform X1 [Plutella xylostella]	Plutella	14	160	3	0.27
1127253092	Aldehyde reductase-1 [Plutella xylostella]	Plutella	1	7	1	0.26
768439087	tumor susceptibility gene 101 protein [Plutella xylostella]	Plutella	4	20	4	0.26
1714823349	uncharacterized protein LOC115446507 [Manduca sexta]	Manduca	1	15	1	0.25
768411909	protein dpy-30 homolog [Plutella xylostella]	Manduca and Plutella	1	14	1	0.25
1714848962	charged multivesicular body protein 4b like [Manduca sexta]	Manduca	3	29	3	0.25
768424285	malignant T-cell-amplified sequence 1 homolog [Plutella xylostella]	Plutella	2	11	2	0.24
1714785891	paxillin isoform X1 [Manduca sexta]	Manduca	3	36	1	0.24
1714812937	vacuolar protein sorting-associated protein 4B [Manduca sexta]	Manduca	6	17	5	0.24
768434562	tumor protein D52 isoform X1 [Plutella xylostella]	Plutella	3	24	2	0.24
768419118	glutenin, high molecular weight subunit 12-like [Plutella xylostella]	Plutella	3	37	3	0.24
1033208529	prophenoloxidase 1 [Plutella xylostella]	Plutella	4	27	2	0.24

1714811156	myophilin [Manduca sexta]	Manduca	2	29	2	0.23
1714842649	GILT-like protein 1 isoform X1 [Manduca sexta]	Manduca	1	1	1	0.23
1714792353	uncharacterized protein LOC115456444 [Manduca sexta]	Manduca	2	5	2	0.23
1714851006	protein DDI1 homolog 2-like isoform X1 [Manduca sexta]	Manduca	1	2	1	0.22
1714802943	troponin T, skeletal muscle isoform X1 [Manduca sexta]	Manduca	19	211	8	0.21
1714793781	target of Myb protein 1 isoform X1 [Manduca sexta]	Manduca	3	6	3	0.21
1714822508	protein yellow-like [Manduca sexta]	Manduca	2	4	2	0.21
768439019	proline synthase co-transcribed bacterial homolog protein [Plutella xylostella]	Plutella	1	2	1	0.21
1714832155	protein DEK [Manduca sexta]	Manduca	4	12	4	0.21
1714812934	U6 snRNA-associated Sm-like protein LSm8 [Manduca sexta]	Manduca	2	4	2	0.20
768419725	LOW QUALITY PROTEIN: rap guanine nucleotide exchange factor 2-like [Plutella xylostella]	Plutella	1	2	1	0.19
1714820485	S-phase kinase-associated protein 1 [Manduca sexta]	Manduca	5	23	5	0.19

**Accession no.**	**Protein**	**Organism**	**# Pept**		**# Unique Peptides**	**Fold-change difference**
1714818997	uncharacterized protein LOC115445531 [Manduca sexta]	Manduca	4	40	1	0.19
1714804609	charged multivesicular body protein 2a [Manduca sexta]	Manduca	2	6	2	0.19
1714810464	uncharacterized protein LOC115443743 isoform X1 [Manduca sexta]	Manduca	4	35	1	0.18
1714824104	hsc70-interacting protein-like [Manduca sexta]	Manduca	1	6	1	0.17
768409364	transcription elongation factor B polypeptide 2 [Plutella xylostella]	Plutella	2	5	1	0.17
768444256	barrier-to-autointegration factor B [Plutella xylostella]	Plutella	3	15	3	0.17
1714844440	ELAV-like protein 1 [Manduca sexta]	Manduca	4	23	4	0.17
1714803042	sorting nexin-12 [Manduca sexta]	Manduca	5	22	5	0.16

1714806639	Y-box factor homolog [Manduca sexta]	Manduca	2	14	1	0.16
768445304	aldehyde dehydrogenase X, mitochondrial like [Plutella xylostella]	Plutella	6	68	1	0.16
1714840381	proteasome subunit beta type-1 [Manduca sexta]	Manduca	3	14	3	0.16
1714826196	serine-protein kinase ATM [Manduca sexta]	Manduca	1	3	1	0.15
1714807269	lysozyme-like [Manduca sexta]	Manduca	1	1	1	0.14
1714835304	protein lethal(2)essential for life-like [Manduca sexta]	Manduca	3	27	1	0.13
1714852506	protein ROP-like [Manduca sexta]	Manduca	5	21	1	0.13
1714826959	poly(U)-binding-splicing factor half pint [Manduca sexta]	Manduca	1	1	1	0.13
1714838135	sorting nexin lst-4 [Manduca sexta]	Manduca	2	7	2	0.13
768435479	digestive cysteine proteinase 2 isoform X1 [Plutella xylostella]	Plutella	3	16	1	0.13
1714850655	pyruvate carboxylase, mitochondrial-like [Manduca sexta]	Manduca	7	43	1	0.12
1714818818	uncharacterized protein LOC115445497 [Manduca sexta]	Manduca	1	2	1	0.11
768420790	gelsolin, partial [Plutella xylostella]	Manduca and Plutella	3	7	3	0.10
768436449	uncharacterized protein LOC105390160 [Plutella xylostella]	Plutella	4	16	4	0.10
768409687	titin [Plutella xylostella]	Plutella	5	15	5	0.09
768435469	coactosin-like protein isoform X1 [Plutella xylostella]	Manduca and Plutella	2	12	2	0.09
1174098354	basic juvenile hormone-suppressible protein 2-like precursor [Plutella xylostella]	Plutella	5	19	4	0.09
1714794490	LOW QUALITY PROTEIN: ubiquilin-1-like [Manduca sexta]	Manduca	6	27	1	0.07
1714820997	thymosin beta isoform X1 [Manduca sexta]	Manduca	4	20	4	0.06
822092754	uncharacterized protein LOC105388666 [Plutella xylostella]	Plutella	18	131	6	0.05
1714835288	heat shock protein 68-like [Manduca sexta]	Manduca	18	129	2	0.05
1714789068	uncharacterized protein LOC115455834 [Manduca sexta]	Manduca	5	31	4	0.04
1714794739	heat shock protein 68-like [Manduca sexta]	Manduca	5	42	2	0.04
1714790048	protein lethal(2)essential for	Manduca	3	9	3	0.04

	life-like [Manduca sexta]					
1714782947	uncharacterized protein LOC115444227 [Manduca sexta]	Manduca	8	20	5	0.03
768417229	heat shock protein 68-like [Plutella xylostella]	Plutella	14	131	1	0.03
1714785079	protein lethal(2)essential for life-like [Manduca sexta]	Manduca	5	35	3	0.02
607029573	small heat shock protein [Plutella xylostella]	Plutella	3	24	2	0.01

Supplementary data file 3. 2-fold change (FC) difference and <0.05 FDR adjusted p-value significance; the 452 proteins were identified as dysregulated from *B. bassiana* infected larvae when compared with the control sample.

Accession	Protein	Organism	# Peptides	# PSMs	# Unique Peptides	Fold change differenc e
768443294	reticulon-1-A isoform X1 [Plutella xylostella]	Plutella	2	12	1	34.30
768431223	LOW QUALITY PROTEIN: interleukin enhancer binding factor 2 homolog [Plutella xylostella]	Plutella	3	10	2	17.15
1714785471	AMP deaminase 2 isoform X1 [Manduca sexta]	Manduca	6	7	1	16.68
1714791788	LOW QUALITY PROTEIN: ryanodine receptor [Manduca sexta]	Manduca	11	26	5	14.95
768431272	calcium uniporter protein, mitochondrial-like isoform X1 [Plutella xylostella]	Manduca and Plutella	1	10	1	10.09
1714841814	26S proteasome non-ATPase regulatory subunit 1 [Manduca sexta]	Manduca	12	68	2	7.72
768442310	40S ribosomal protein S2 [Plutella xylostella]	Plutella	14	151	1	7.39
1714843099	neurochondrin homolog [Manduca sexta]	Manduca	5	17	1	7.38
768428731	uncharacterized protein LOC105386610 [Plutella xylostella]	Manduca and Plutella	1	10	1	7.31
768439318	V-type proton ATPase 116 kDa subunit a isoform 1 isoform X3 [Plutella xylostella]	Plutella	12	60	2	7.27

768432945	extended synaptotagmin-2-A-like, partial [Plutella xylostella]	Plutella	8	27	2	6.83
768427005	uncharacterized protein LOC105385824 [Plutella xylostella]	Plutella	5	21	2	6.72
768442606	vesicle-trafficking protein SEC22b [Plutella xylostella]	Plutella	4	9	4	6.71
1714820720	vacuolar protein sorting-associated protein 35 isoform X1 [Manduca sexta]	Manduca	2	9	2	6.63
768410384	translational activator GCN1-like [Plutella xyl		5	16	5	6.30
768429978	programmed cell death protein 6 isoform X1 [Plutella xylostella]	Plutella	3	13	3	6.04
768442023	ATP-dependent RNA helicase WM6 [Plutella xylostella]	Plutella	12	53	1	6.02
1714837734	innexin inx2 [Manduca sexta]	Manduca	2	8	2	5.36
1714843767	protein transport protein Sec61 subunit alpha [Manduca sexta]	Manduca	9	68	9	5.31
1194641147	phosphate carrier 1 [Plutella xylostella]	Plutella	6	62	1	5.24
1714793391	importin-7 isoform X1 [Manduca sexta]	Manduca	4	8	4	5.09
1127252922	UDP-glycosyltransferase-42F1 [Plutella xylostella]	Plutella	1	3	1	5.04
768439702	nucleolar protein 56-like [Plutella xylostella]	Plutella	5	25	1	4.96
1714843905	lysine--tRNA ligase isoform X1 [Manduca sexta]	Manduca	5	40	2	4.88
1714845569	eukaryotic translation initiation factor 3 subunit K [Manduca sexta]	Manduca	7	26	6	4.76
768445241	heterogeneous nuclear ribonucleoprotein R-like [Plutella xylostella]	Plutella	11	51	2	4.57
768415447	calcium-transporting ATPase sarcoplasmic/endoplasmic reticulum type isoform X1 [Plutella xylostella]	Plutella	21	229	2	4.56
768414774	V-type proton ATPase subunit H isoform X1 [Plutella xylostella]	Plutella	16	92	2	4.55
768413506	neurochondrin homolog [Plutella xylostella]	Plutella	5	23	1	4.54
1714839839	UDP-glucuronosyltransferase 2B4-like [Manduca sexta]	Manduca	2	10	1	4.47
1714836771	aromatic-L-amino-acid decarboxylase [Manduca sexta]	Manduca	2	12	1	4.40

Accession	Protein	Organism	# Peptides	# PSMs	# Unique Peptides	Fold change difference
1714810738	mitochondrial carrier homolog 2-like [Manduca sexta]	Manduca	2	12	2	4.35
768441612	spectrin alpha chain-like, partial [Plutella xylostella]	Plutella	40	291	1	4.34
768443899	programmed cell death protein 4-like [Plutella xylostella]	Manduca and Plutella	2	8	2	4.27
1714824983	synaptic vesicle glycoprotein 2C-like isoform X1 [Manduca sexta]	Manduca	1	6	1	4.25
1714807772	integrin beta-PS [Manduca sexta]	Manduca	2	6	2	4.24
1714829531	dolichyl-diphosphooligosaccharide--protein glycosyltransferase subunit STT3A [Manduca sexta]	Manduca	4	13	4	4.13
1714802768	V-type proton ATPase 16 kDa proteolipid subunit [Manduca sexta]	Manduca	2	36	2	4.10
1714782743	long-chain-fatty-acid--CoA ligase 4 isoform X1 [Manduca sexta]	Manduca	4	40	2	4.08
1714847100	UDP-glucuronosyltransferase 2B4-like isoform X1 [Manduca sexta]	Manduca	3	12	2	4.07
768419379	muscle M-line assembly protein unc-89-like [Plutella xylostella]	Plutella	9	42	4	4.07
768445010	clathrin heavy chain isoform X1 [Plutella xylostella]	Plutella	33	177	3	4.06
1714837777	116 kDa U5 small nuclear ribonucleoprotein component [Manduca sexta]	Manduca	5	10	5	4.01
1714795408	cAMP-dependent protein kinase catalytic subunit 1 [Manduca sexta]	Manduca	7	19	7	3.80
165932398	cytochrome c oxidase subunit II (mitochondrion) [Manduca sexta]	Manduca	5	35	1	3.78
1714786609	probable N-acetyltransferase san [Manduca sexta]	Manduca	2	5	1	3.77
1714825426	importin-5 [Manduca sexta]	Manduca	9	51	9	3.73
768449720	coatomer subunit beta-like [Plutella xylostella]	Plutella	5	16	4	3.71
1714806575	eukaryotic translation initiation factor 4	Manduca	3	10	3	3.69

	gamma 3- like isoform X1 [Manduca sexta]					
1714816033	cullin-associated NEDD8-dissociated protein 1 [Manduca sexta]	Manduca	7	14	3	3.61
1714787824	Krueppel homolog 2 [Manduca sexta]	Manduca	1	7	1	3.60
1714784318	ATP synthase lipid-binding protein, mitochondrial [Manduca sexta]	Manduca	1	39	1	3.59
1714851726	protein krasavietz [Manduca sexta]	Manduca	5	14	5	3.59
1714854095	phosphate carrier protein, mitochondrial-like [Manduca sexta]	Manduca	6	53	1	3.56
768417308	sodium/potassium-transporting ATPase subunit beta-2 [Plutella xylostella]	Manduca and Plutella	2	18	2	3.55
768438622	calcium/calmodulin-dependent protein kinase type II alpha chain [Plutella xylostella]	Plutella	5	26	1	3.50
1714836738	V-type proton ATPase subunit d [Manduca sexta]	Manduca	5	18	2	3.45
768446145	FUN14 domain-containing protein 1-like isoform X1 [Plutella xylostella]	Manduca and Plutella	1	4	1	3.44
768421353	glycogen [starch] synthase isoform X1 [Plutella xylostella]	Plutella	2	19	2	3.42
1714819420	D-beta-hydroxybutyrate dehydrogenase, mitochondrial [Manduca sexta]	Manduca	6	46	2	3.41

**Accession**	**Protein**	**Organism**	**# Peptides**	**# PSMs**	**# Unique Peptides**	**Fold change difference**
1714816610	glutamine--fructose-6-phosphate aminotransferase [isomerizing] 1-like [Manduca sexta]	Manduca	4	14	4	3.39
1714786780	protein phosphatase 1A [Manduca sexta]	Manduca	4	11	4	3.36
1714812709	coatomer subunit alpha isoform X2 [Manduca sexta]	Manduca	14	28	3	3.34
768430159	arginine--tRNA ligase, cytoplasmic [Plutella xylostella]	Plutella	4	20	1	3.33
1714815689	beta-parvin [Manduca sexta]	Manduca	3	14	1	3.32

768415371	sodium/potassium-transporting ATPase subunit alpha isoform X1 [Plutella xylostella]	Plutella	29	148	29	3.30
1714798516	uncharacterized protein LOC115441016 [Manduca sexta]	Manduca	13	96	13	3.26
768432042	1,4-alpha-glucan-branching enzyme isoform X2 [Plutella xylostella]	Plutella	5	17	2	3.25
787071490	adenine nucleotide translocase insect3 [Plutella xylostella]	Plutella	16	203	2	3.25
1714832549	LOW QUALITY PROTEIN: multidrug resistance protein homolog 49-like [Manduca sexta]	Manduca	3	8	3	3.22
768421373	glutamate synthase [NADH], amyloplastic-like [Plutella xylostella]	Plutella	7	20	3	3.21
1714845899	proline dehydrogenase 1, mitochondrial-like [Manduca sexta]	Manduca	5	44	5	3.21
768438263	myosin-IB-like [Plutella xylostella]	Plutella	2	4	2	3.19
1714808825	serine/threonine-protein phosphatase 2A 56 kDa regulatory subunit epsilon isoform [Manduca sexta Manduca		5	16	5	3.18
1714844215	tubulin beta chain-like [Manduca sexta]	Manduca	14	118	3	3.17
1714818345	alpha-aminoadipic semialdehyde synthase, mitochondrial [Manduca sexta]	Manduca	12	80	3	3.15
768408627	apolipophorins-like [Plutella xylostella]	Plutella	8	93	6	3.14
768435072	mitochondrial import inner membrane translocase subunit Tim17-B [Plutella xylostella]	Manduca and Plutella	2	3	2	3.12
1714852402	la-related protein 1-like, partial [Manduca sexta]	Manduca	2	10	2	3.12
768440535	laminin subunit alpha-like, partial [Plutella xylostella]	Plutella	6	30	2	3.11
1714824089	piwi-like protein Siwi [Manduca sexta]	Manduca	7	24	5	3.09
768433865	eukaryotic translation initiation factor 3 subunit B [Plutella xylostella]	Plutella	12	46	12	3.08
671871149	P450 CYP6 family protein 6 [Plutella xylostella]	Plutella	1	11	1	3.07
768428328	puromycin-sensitive aminopeptidase isoform X1 [Plutella xylostella]	Plutella	6	30	2	3.06
1714830135	probable aminopeptidase NPEPL1 isoform X1 [Manduca sexta]	Manduca	2	11	2	3.06

768436859	kynurenine 3-monooxygenase-like [Plutella xylostella]	Plutella	2	6	2	3.05
1714854058	protein MEMO1 [Manduca sexta]	Manduca	3	9	3	3.04
1714798431	uncharacterized protein LOC115440997 isoform X1 [Manduca sexta]	Manduca	11	102	3	3.00
1714780065	translocating chain-associated membrane protein 1 [Manduca sexta]	Manduca	1	9	1	2.92
768416529	tryptophan 2,3-dioxygenase-like [Plutella xyl		3	15	3	2.92
768408066	hexokinase type 2 isoform X1 [Plutella xylostella]	Plutella	10	32	2	2.90

**Accession**	**Protein**	**Organism**	**# Peptides**	**# PSMs**	**# Unique Peptides**	**Fold change difference**
768439997	lysine--tRNA ligase isoform X1 [Plutella xylostella]	Plutella	5	46	2	2.88
768414045	S-formylglutathione hydrolase [Plutella xylostella]	Plutella	3	25	3	2.86
1714784806	LOW QUALITY PROTEIN: tripeptidyl-peptidase 2 [Manduca sexta]	Manduca	2	13	2	2.85
1714793124	von Willebrand factor A domain-containing protein 8 [Manduca sexta]	Manduca	5	11	4	2.84
1714817478	translocon-associated protein subunit gamma [Manduca sexta]	Manduca	2	19	2	2.83
1714790982	NADH-ubiquinone oxidoreductase 49 kDa subunit [Manduca sexta]	Manduca	8	45	8	2.80
768441273	scavenger receptor class B member 1 isoform X1 [Plutella xylostella]	Manduca and Plutella	2	7	2	2.79
768426118	regulator of nonsense transcripts 1-like [Plutella xylostella]	Manduca and Plutella	5	13	5	2.79
1714797205	ubiquitin carboxyl-terminal hydrolase 7-like isoform X1 [Manduca sexta]	Manduca	11	50	7	2.78
1714795881	protein sly1 homolog [Manduca sexta]	Manduca	2	10	2	2.78
1714824281	1,4-alpha-glucan-branching enzyme [Manduca sexta]	Manduca	5	22	2	2.77
1714835219	serine/threonine-protein phosphatase 2B catalytic subunit 3-like [Manduca sexta]	Manduca	10	25	10	2.76

1714820011	myosin heavy chain 95F [Manduca sexta]	Manduca	1	8	1	2.73
1714830307	threonine--tRNA ligase, cytoplasmic isoform X1 [Manduca sexta]	Manduca	11	52	11	2.71
768426554	mitochondrial import inner membrane translocase subunit TIM50-C-like isoform X1 [Plutella xylostella Plutella		3	6	3	2.69
1714798572	protein transport protein Sec61 subunit beta [Manduca sexta]	Manduca	1	9	1	2.69
1714808308	clathrin heavy chain [Manduca sexta]	Manduca	40	202	10	2.67
768438040	ATP-binding cassette sub-family E member 1 [Plutella xylostella]	Plutella	8	44	8	2.66
1714836224	ATP synthase subunit gamma, mitochondrial [Manduca sexta]	Manduca	3	40	2	2.66
1714796960	tolloid-like protein 1 [Manduca sexta]	Manduca	1	8	1	2.66
768439586	calcium-binding mitochondrial carrier protein Aralar1-like [Plutella xylostella]	Plutella	3	23	1	2.65
768420963	LOW QUALITY PROTEIN: stomatin-like protein 2, mitochondrial [Plutella xylostella]	Plutella	3	13	1	2.64
1714779918	casein kinase II subunit alpha isoform X1 [Manduca sexta]	Manduca	8	17	8	2.63
768407834	transmembrane emp24 domain-containing protein bai [Plutella xylostella]	Plutella	4	36	4	2.62
768419761	protein phosphatase 1L [Plutella xylostella]	Plutella	1	1	1	2.62
1714790494	glutaryl-CoA dehydrogenase, mitochondrial [Manduca sexta]	Manduca	2	10	2	2.62
1714824402	nucleolar protein 58 [Manduca sexta]	Manduca	6	27	2	2.62
1714813393	arginine--tRNA ligase, cytoplasmic [Manduca sexta]	Manduca	4	14	1	2.61
768425216	eukaryotic translation initiation factor 3 subunit K [Plutella xylostella]	Plutella	4	9	3	2.61

**Accession**	**Protein**	**Organism**	**# Peptides**	**# PSMs**	**# Unique Peptides**	**Fold change difference**
1714796863	calcium-binding mitochondrial carrier protein Aralar1 isoform X1 [Manduca sexta]	Manduca	7	40	5	2.60

1714789907	plasma membrane calcium-transporting ATPase 2 isoform X1 [Manduca sexta]	Manduca	25	139	25	2.59
1714858387	ADP,ATP carrier protein [Manduca sexta]	Manduca	16	204	2	2.59
1714814980	lon protease homolog, mitochondrial isoform X1 [Manduca sexta]	Manduca	6	29	6	2.58
1714843560	cytochrome P450 9e2-like [Manduca sexta]	Manduca	3	19	3	2.57
768411720	2-oxoglutarate dehydrogenase, mitochondrial [Plutella xylostella]	Plutella	28	289	1	2.54
1714836369	ATP-citrate synthase [Manduca sexta]	Manduca	39	325	14	2.54
1714817964	cyclin-L1 [Manduca sexta]	Manduca	1	4	1	2.52
1714817988	cytochrome c oxidase subunit 4 isoform 1, mitochondrial-like [Manduca sexta]	Manduca	2	12	2	2.52
768415174	probable 26S proteasome non-ATPase regulatory subunit 3 [Plutella xylostella]	Plutella	8	30	2	2.51
1714854061	nicotinate phosphoribosyltransferase isoform X1 [Manduca sexta]	Manduca	1	9	1	2.48
1714838480	metal transporter CNNM4-like [Manduca sexta]	Manduca	5	27	5	2.47
1714831683	uncharacterized protein LOC115448315 isoform X1 [Manduca sexta]	Manduca	2	2	2	2.46
1714816594	TAR DNA-binding protein 43-like [Manduca sexta]	Manduca	2	3	2	2.45
1714835669	dynamin-like 120 kDa protein, mitochondrial isoform X1 [Manduca sexta]	Manduca	6	11	6	2.44
1127252882	Fatty acid synthase-4, partial [Plutella xylostella]	Plutella	3	35	1	2.44
1714787675	amino acid transporter AVT1B-like [Manduca sexta]	Manduca	1	7	1	2.44
1714795264	PTB domain-containing adapter protein ced-6 isoform X1 [Manduca sexta]	Manduca	2	5	1	2.44
1714794110	puromycin-sensitive aminopeptidase [Manduca sexta]	Manduca	10	40	6	2.43
1714858948	dolichyl-diphosphooligosaccharide--protein glycosyltransferase subunit 1, partial [Manduca sexta]	Manduca	6	48	6	2.42
768410921	tRNA-splicing ligase RtcB homolog [Plutella xylostella]	Plutella	2	6	2	2.41
768429473	D-beta-hydroxybutyrate dehydrogenase, mitochondrial isoform X1 [Plutella	Plutella	5	27	1	2.41

	xylostella]					
768423690	bifunctional glutamate/proline--tRNA ligase isoform X1 [Plutella xylostella]	Plutella	7	34	6	2.40
768444918	clustered mitochondria protein homolog [Plutella xylostella]	Plutella	7	19	2	2.40
1714840384	dolichyl-diphosphooligosaccharide--protein glycosyltransferase subunit DAD1 [Manduca sexta]	Manduca	1	1	1	2.39
1714816829	E3 SUMO-protein ligase RanBP2-like [Manduca sexta]	Manduca	1	4	1	2.37
768433466	myosin heavy chain, muscle isoform X9 [Plutella xylostella]	Plutella	160	2817	1	2.36
1714799853	prolyl endopeptidase isoform X1 [Manduca sexta]	Manduca	6	17	6	2.35
768410047	uncharacterized protein LOC105387140 isoform X1 [Plutella xylostella]	Plutella	10	100	2	2.35

Accession	Protein	Organism	# Peptides	# PSMs	# Unique Peptides	Fold change difference
1714789770	AFG3-like protein 2 [Manduca sexta]	Manduca	13	41	3	2.34
1714841515	reticulon-1 isoform X1 [Manduca sexta]	Manduca	2	9	1	2.34
768421422	probable galactose-1-phosphate uridylyltransferase [Plutella xylostella]	Plutella	2	5	2	2.33
1714782216	glycogen synthase kinase-3 beta-like isoform X1 [Manduca sexta]	Manduca	4	11	4	2.31
1714822723	acetyl-CoA carboxylase isoform X1 [Mandu		34	186	19	2.31
768409171	LOW QUALITY PROTEIN: talin-1-like, partial [Plutella xylostella]	Plutella	2	13	1	2.30
1714811114	chloride intracellular channel exc-4 isoform X1 [Manduca sexta]	Manduca	5	33	5	2.30
1714819901	FK506-binding protein 5 isoform X5 [Manduca sexta]	Manduca	12	112	2	2.30
1714786519	alpha-actinin, sarcomeric isoform X1 [Manduca sexta]	Manduca	61	664	16	2.30
1714812923	NADP-dependent malic enzyme-like isoform	Manduca	10	58	10	2.29

	X1 [Manduca sexta]					
768407893	presequence protease, mitochondrial-like [Plutella xylostella]	Plutella	3	9	2	2.29
1714827928	AP-2 complex subunit mu [Manduca sexta]	Manduca	7	15	7	2.29
1714815611	dipeptidyl peptidase 3 isoform X1 [Manduca sexta]	Manduca	3	25	1	2.28
768431420	NADH dehydrogenase [ubiquinone] 1 beta subcomplex subunit 5, mitochondrial-like [Plutella xylostella]	Plutella	1	4	1	2.27
488444153	serine/threonine-protein phosphatase 2A catalytic subunit [Plutella xylostella]	Manduca and Plutella	5	26	5	2.26
1714834348	probable glutamine--tRNA ligase [Manduca sexta]	Manduca	4	21	1	2.25
768429249	LOW QUALITY PROTEIN: 26S proteasome non ATPa regulatory subunit 1-like [Plutella xylostella] Plutell		12	64	2	2.24
1714844507	endocuticle structural glycoprotein SgAbd-8-like [Manduca sexta]	Manduca	3	7	3	2.24
768436004	plectin-like [Plutella xylostella]	Plutella	5	16	1	2.21
768437714	T-complex protein 1 subunit eta [Plutella xyl		14	82	5	2.21
1714825279	28S ribosomal protein S5, mitochondrial [Manduca sexta]	Manduca	2	5	2	2.20
768421658	guanine nucleotide-binding protein G(o) subunit alpha isoform X1 [Plutella xylostella]	Manduca and Plutella	8	34	8	2.20
1714839511	putative tricarboxylate transport protein, mitochondrial [Manduca sexta]	Manduca	5	32	5	2.20
1714823275	putative ATP synthase subunit f, mitochondrial [Manduca sexta]	Manduca	1	18	1	2.20
1714841381	ADP-ribosylation factor-like protein 8 [Manduca sexta]	Manduca	3	7	3	2.19
768448059	coatomer subunit beta'-like [Plutella xylostella]	Plutella	5	17	3	2.18
768415199	26S proteasome non-ATPase regulatory subunit 6 [Plutella xylostella]	Plutella	3	8	1	2.18
1714824678	adenylosuccinate synthetase [Manduca sexta]	Manduca	3	12	3	2.17
768408615	alpha-actinin, sarcomeric-like [Plutella xylostella]	Plutella	50	555	5	2.17

Accession	Protein	Organism	# Peptides	# PSMs	# Unique Peptides	Fold change difference
1714804170	calcium-transporting ATPase sarcoplasmic/endoplasmic reticulum type isoform X1 [Manduca sexta]	Manduca	26	266	7	2.16
768422071	plastin-3 isoform X3 [Plutella xylostella]	Manduca and Plutella	8	38	7	2.16
1714845965	cAMP-dependent protein kinase type I regulatory subunit isoform X1 [Manduca sexta]	Manduca	5	18	5	2.15
1714842399	asparagine--tRNA ligase, cytoplasmic [Manduca sexta]	Manduca	2	9	2	2.14
768412475	rab GDP dissociation inhibitor alpha [Plutella xylostella]	Plutella	16	90	7	2.14
1714791392	isocitrate dehydrogenase [NAD] subunit gamma, mitochondrial-like [Manduca sexta]	Manduca	2	28	2	2.13
768408999	NADH dehydrogenase [ubiquinone] iron-sulfur protein 3, mitochondrial-like [Plutella xylostella]	Plutella	9	44	9	2.12
1714790005	twitchin isoform X1 [Manduca sexta]	Manduca	74	411	44	2.11
1714837691	mitochondrial import receptor subunit TOM40 homolog 1-like [Manduca sexta]	Manduca	4	38	4	2.10
1714821287	collagen alpha-1(IV) chain [Manduca sexta]	Manduca	2	26	2	2.10
1714823704	dolichyl-diphosphooligosaccharide--protein glycosyltransferase subunit 2 [Manduca sexta]	Manduca	2	10	2	2.09
1714794307	dynein heavy chain, cytoplasmic isoform X1 [Manduca sexta]	Manduca	23	57	23	2.09
1714795867	xanthine dehydrogenase [Manduca sexta]	Manduca	4	20	4	2.09
1714854521	polyadenylate-binding protein-interacting protein 1 [Manduca sexta]	Manduca	1	4	1	2.08
768443210	tripeptidyl-peptidase 2 [Plutella xylostella]	Plutella	2	6	2	2.08
1714784562	60S ribosomal protein L15 [Manduca sexta]	Manduca	8	64	8	2.08
1714806087	ras-related protein Rac1 [Manduca sexta]	Manduca	4	13	4	2.07
768429300	T-complex protein 1 subunit zeta	Plutella	6	53	1	2.06

	[Plutella xylostella]					
1714847173	LOW QUALITY PROTEIN: glutamate synthase [NADH], amyloplastic [Manduca sexta]	Manduca	14	60	10	2.06
1127252884	Fatty acid synthase-5 [Plutella xylostella]	Plutella	2	11	1	2.05
1714825936	ubiquitin-like modifier-activating enzyme 1 [Manduca sexta]	Manduca	8	54	4	2.04
768417356	T-complex protein 1 subunit epsilon [Plutella xylostella]	Plutella	9	59	4	2.02
768438558	glucose-6-phosphate isomerase [Plutella xylostella]	Plutella	6	68	2	2.02
768418411	2-hydroxyacyl-CoA lyase 1-like isoform X1 [Plutella xylostella]	Plutella	1	8	1	2.02
1714798768	phosphoribosylformylglycinamidine synthase [Manduca sexta]	Manduca	10	103	6	2.02
768430014	alanine--tRNA ligase, cytoplasmic [Plutella xylostella]	Plutella	4	22	3	2.02
768424454	V-type proton ATPase subunit C isoform X1 [Plutella xylostella]	Plutella	11	77	3	2.01
1714847252	serine/threonine-protein phosphatase 2A 65 kDa regulatory subunit A alpha isoform [Manduca sexta Manduca		14	82	4	2.01
1714788345	ATP-dependent RNA helicase WM6 [Manduca sexta]	Manduca	18	88	7	2.01

Accession	Protein	Organism	# Peptides	# PSMs	# Unique Peptides	Fold change difference
768433032	CCHC-type zinc finger protein CG3800 [Plutella xylostella]	Plutella	2	19	2	2.00
1714814714	uncharacterized protein LOC115444704, partial [Manduca sexta]	Manduca	51	186	34	2.00
1714800092	ubiquitin-conjugating enzyme E2 L3 [Manduca sexta]	Manduca	7	50	7	0.50
1714812765	probable small nuclear ribonucleoprotein Sm D2 [Manduca sexta]	Manduca	7	51	7	0.50
768419924	non-specific lipid-transfer protein	Plutella	7	129	3	0.50

	[Plutella xylostella]					
768447461	myosin regulatory light chain 2-like [Plutella xylostella]	Plutella	4	108	4	0.50
768423176	neurogenic locus notch homolog protein 4-like [Plutella xylostella]	Plutella	2	21	1	0.50
768446070	uncharacterized protein LOC105394749 [Plutella xylostella]	Plutella	1	1	1	0.49
1714829845	SUMO-conjugating enzyme UBC9-A [Manduca sexta]	Manduca and Plutella	8	32	8	0.49
388252732	beta-1,3-glucan binding protein [Plutella xylostella]	Plutella	1	6	1	0.49
768427525	homogentisate 1,2-dioxygenase isoform X1 [Plutella xylostella]	Plutella	2	9	2	0.49
768411878	tyrosine--tRNA ligase, cytoplasmic [Plutella xylostella]	Plutella	4	25	1	0.49
1714828540	peroxiredoxin [Manduca sexta]	Manduca	4	30	3	0.49
768434824	dnaJ homolog subfamily C member 3 [Plutella xylostella]	Manduca and Plutella	1	5	1	0.49
1714788166	septin-1 [Manduca sexta]	Manduca	1	2	1	0.49
768444929	adenylate kinase-like [Plutella xylostella]	Plutella	3	26	1	0.49
768443464	DNA-binding protein DDB_G0278111 [Plutella xylostella]	Plutella	1	5	1	0.49
770075643	60S acidic ribosomal protein P1 [Plutella xylostella]	Plutella	3	245	3	0.49
768445002	ran-specific GTPase-activating protein-like [Plutella xylostella]	Manduca and Plutella	1	3	1	0.49
1714798945	lambda-crystallin homolog [Manduca sexta]	Manduca	2	6	1	0.49
768431748	heterogeneous nuclear ribonucleoprotein K [Plutella xylostella]	Manduca and Plutella	2	10	2	0.49
1714819213	heterogeneous nuclear ribonucleoprotein A1-like [Manduca sexta]	Manduca	3	23	3	0.49
768434394	calcyphosin-like protein isoform X1 [Plutella xylostella]	Plutella	1	2	1	0.48
1714822949	protein dj-1beta-like [Manduca sexta]	Manduca	1	3	1	0.48

1714844700	aldo-keto reductase family 1 member B1-like isoform X1 [Manduca sexta]	Manduca	6	72	5	0.48
1714830944	UV excision repair protein RAD23 homolog A [Manduca sexta]	Manduca	5	20	5	0.48
768437730	delta-1-pyrroline-5-carboxylate synthase isoform X1 [Plutella xylostella]	Plutella	7	62	2	0.48
1714833458	40S ribosomal protein S24 [Manduca sexta]	Manduca	6	96	6	0.48
1714806091	60S ribosomal protein L22-like [Manduca sexta]	Manduca	1	29	1	0.48
1714809097	troponin C, isoallergen Bla g 6.0301-like isoform X1 [Manduca sexta]	Manduca	15	274	10	0.48
1714799207	catalase [Manduca sexta]	Manduca	16	201	9	0.48

Accession	Protein	Organism	# Peptides	# PSMs	# Unique Peptides	Fold change difference
768418361	serine hydroxymethyltransferase, cytosolic [Plutella xylostella]	Plutella	3	25	2	0.48
1714822427	angiotensin-converting enzyme-like isoform X1 [Manduca sexta]	Manduca	2	6	2	0.47
1714834544	apolipophorin-3 [Manduca sexta]	Manduca	1	27	1	0.47
768410288	macrophage migration inhibitory factor-like [Plutella xylostella]	Plutella	2	21	2	0.47
1714851006	protein DDI1 homolog 2-like isoform X1 [Manduca sexta]	Manduca	1	2	1	0.47
1714826809	cuticle protein 3-like [Manduca sexta]	Manduca	2	15	2	0.47
1714818638	tumor protein D54 isoform X1 [Manduca sexta]	Manduca	4	26	3	0.47
768447965	heat shock protein beta-1-like [Plutella xylostella]	Plutella	8	63	8	0.47
1714804878	lipoamide acyltransferase component of branched chain alpha-keto acid dehydrogenase complex, mitochondrial [Manduca sexta]	Manduca	3	15	3	0.47
768415298	GTP-binding protein SAR1b-like [Plutella xylostella]	Plutella	5	41	3	0.47
1714802943	troponin T, skeletal muscle isoform X1 [Manduca sexta]	Manduca	19	211	8	0.46
768424763	protein disulfide-isomerase-like [Plutella xylostella]	Plutella	5	59	1	0.46

1714820457	succinate dehydrogenase assembly factor 2-A, mitochondrial-like isoform X1 [Manduca sexta]	Manduca	1	7	1	0.46
1714829415	NHP2-like protein 1 [Manduca sexta]	Manduca	1	2	1	0.46
768413904	heat shock 70 kDa protein cognate 3-like [Plutella xylostella]	Plutella	33	480	5	0.46
1714818034	uncharacterized protein PB18E9.04c [Manduca sexta]	Manduca	1	7	1	0.46
768425749	muscle-specific protein 20 [Plutella xylostella]	Plutella	2	74	2	0.46
768420285	catalase-like [Plutella xylostella]	Plutella	11	155	4	0.46
1714843350	tropomyosin-2 isoform X7 [Manduca sexta]	#N/A	29	533	0	0.46
1714795060	translation machinery-associated protein 7 homolog isoform X2 [Manduca sexta]	Manduca	3	9	3	0.46
768426258	ras-related protein Rab-32 isoform X1 [Plutella xylostella]	Manduca and Plutella	2	2	2	0.46
1714801062	rho GDP-dissociation inhibitor 1 isoform X2 [Manduca sexta]	Manduca	5	33	5	0.45
1714811260	aldehyde dehydrogenase X, mitochondrial-like [Manduca sexta]	Manduca	7	62	2	0.45
768410072	actin-related protein 2/3 complex subunit 5 [Plutella xylostella]	Plutella	2	4	2	0.45
768408131	uncharacterized protein ZC395.10-like [Plutella xylostella]	Manduca and Plutella	1	5	1	0.45
1714846702	calcineurin B homologous protein 1 [Manduca sexta]	Manduca	3	6	3	0.45
1714819169	heterogeneous nuclear ribonucleoprotein 87F-like [Manduca sexta]	Manduca	4	35	2	0.45
1714799293	nucleobindin-2 isoform X1 [Manduca sexta]	Manduca	4	12	4	0.45
768438732	U1 small nuclear ribonucleoprotein C [Plutella xylostella]	Manduca and Plutella	2	9	2	0.45
1714781486	DDRGK domain-containing protein 1 [Manduca sexta]	Manduca	1	8	1	0.45

Accession	Protein	Organism	# Peptides	# PSMs	# Unique Peptides	Fold change difference
1714816672	extended synaptotagmin-2-A isoform X1 [Manduca sexta]	Manduca	9	35	3	0.45
1714827067	E3 ubiquitin-protein ligase KCMF1, partial [Manduca sexta]	Manduca	1	12	1	0.45
1714784627	eukaryotic translation initiation factor 3 subunit A [Manduca sexta]	Manduca	1	10	1	0.45
768425078	transmembrane protease serine 9-like [Plutella xylostella]	Manduca and Plutella	1	5	1	0.45
1714803645	FK506-binding protein 59 isoform X1 [Manduca sexta]	Manduca	2	22	2	0.45
1714811156	myophilin [Manduca sexta]	Manduca	2	29	2	0.44
1714834998	poly(U)-specific endoribonuclease homolog [Manduca sexta]	Manduca	1	1	1	0.44
1714780838	glycine-rich cell wall structural protein [Manduca sexta]	Manduca	2	32	2	0.44
1714800745	protein disulfide-isomerase A3 [Manduca sexta]	Manduca	3	32	2	0.44
1714811257	aldehyde dehydrogenase X, mitochondrial-like [Manduca sexta]	Manduca	8	69	3	0.44
768415229	alpha-amylase 4N-like [Plutella xylostella]	Plutella	1	14	1	0.44
1714780595	alpha-amylase 4N-like isoform X1 [Manduca sexta]	Manduca	1	6	1	0.44
768448326	probable prefoldin subunit 4 [Plutella xylostella]	Plutella	1	10	1	0.44
1714814635	nuclear migration protein nudC [Manduca sexta]	Manduca	5	29	5	0.44
768425405	uncharacterized protein LOC105385083 [Plutella xylostella]	Plutella	1	1	1	0.44
768435479	digestive cysteine proteinase 2 isoform X1 [Plutella xylostella]	Plutella	3	16	1	0.43
1714819196	prefoldin subunit 6 [Manduca sexta]	Manduca	5	32	5	0.43
1714783673	uncharacterized protein LOC115445754 isoform X1 [Manduca sexta]	Manduca	1	10	1	0.43
768436581	arginine kinase isoform X1 [Plutella xylostella]	Plutella	21	1095	1	0.43
768419735	troponin C, isoform 3-like [Plutella xylostella]	Plutella	9	118	4	0.42

Accession	Protein	Organism	# Peptides	# PSMs	# Unique Peptides	Fold change difference
1714847005	delta(3,5)-Delta(2,4)-dienoyl-CoA isomerase, mitochondrial-like isoform X1 [Manduca sexta]	Manduca	1	6	1	0.42
768436047	aldose reductase-like [Plutella xylostella]	Plutella	2	16	1	0.42
1714854753	phenoloxidase-activating factor 2-like, partial [Manduca sexta]	Manduca	2	18	2	0.42
1714793760	protein FAM49B [Manduca sexta]	Manduca	1	6	1	0.42
768430566	THO complex subunit 4 [Plutella xylostella]	Plutella	1	4	1	0.42
1714840571	serine/threonine-protein kinase minibrain-like [Manduca sexta]	Manduca	1	5	1	0.41
1714820008	myophilin-like [Manduca sexta]	Manduca	3	24	2	0.41
768420532	tropomyosin-2 isoform X2 [Plutella xylostella]	Plutella	20	368	2	0.41
1714832438	28S ribosomal protein S17, mitochondrial [Manduca sexta]	Manduca	2	6	2	0.41
768443164	flavin reductase (NADPH) [Plutella xylostella]	Plutella	1	12	1	0.41
1714840539	calreticulin [Manduca sexta]	Manduca	8	92	4	0.41
1714800977	histone H3.3 [Manduca sexta]	Manduca and Plutella	5	73	1	0.41
1714845982	general transcription factor IIF subunit 1-like [Manduca sexta]	Manduca	4	23	4	0.41
768447528	sorting nexin-2-like [Plutella xylostella]	Plutella	3	7	2	0.41
1714842649	GILT-like protein 1 isoform X1 [Manduca sexta]	Manduca	1	1	1	0.40

Accession	Protein	Organism	# Peptides	# PSMs	# Unique Peptides	Fold change differenc e
1714804210	troponin C, isoallergen Bla g 6.0101-like [Manduca sexta]	Manduca	1	2	1	0.40
768437513	protein held out wings [Plutella xylostella]	Plutella	3	6	3	0.40
768412686	microtubule-associated protein RP/EB family member 3, partial [Plutella xylostella]	Plutella	3	27	2	0.40
1714815968	sialic acid synthase [Manduca sexta]	Manduca	4	21	2	0.40
1714792573	putative aminopeptidase W07G4.4 [Mandu		2	10	1	0.39

1714818997	uncharacterized protein LOC115445531 [Manduca sexta]	Manduca	4	40	1	0.39
768428921	mRNA-decapping enzyme 1B-like [Plutella xylostella]	Manduca and Plutella	2	7	2	0.39
768408976	protein D2-like isoform X1 [Plutella xylostella]	Plutella	4	27	4	0.39
1714802920	glycine N-methyltransferase isoform X1 [Manduca sexta]	Manduca	4	38	4	0.39
768449175	hydroxyacyl-coenzyme A dehydrogenase, mitochondrial-like [Plutella xylostella]	Plutella	5	44	1	0.39
1714846630	aldo-keto reductase family 1 member B1-like [Manduca sexta]	Manduca	5	51	3	0.38
1714829323	uncharacterized protein LOC115447784 isoform X1 [Manduca sexta]	Manduca	1	2	1	0.38
1714799300	chitinase-like protein EN03 isoform X1 [Manduca sexta]	Manduca	4	186	4	0.38
1714798738	uncharacterized protein LOC115441057 [Manduca sexta]	Manduca	1	15	1	0.38
768411704	SH3 domain-binding glutamic acid-rich protein homolog [Plutella xylostella]	Plutella	2	12	2	0.38
1714805237	uncharacterized protein LOC115442608 isoform X1 [Manduca sexta]	Manduca	2	3	2	0.38
1714830170	triosephosphate isomerase [Manduca sexta]	Manduca	7	93	3	0.38
1714803042	sorting nexin-12 [Manduca sexta]	Manduca	5	22	5	0.37
768434790	fructose-bisphosphate aldolase-like, partial [Plutella xylostella]	Plutella	1	27	1	0.37
1714823667	acyl carrier protein 1, mitochondrial isoform X2 [Manduca sexta]	Manduca	1	8	1	0.37
768415052	mesencephalic astrocyte-derived neurotrophic factor homolog [Plutella xylostella]	Manduca and Plutella	1	3	1	0.37
1714781802	calmodulin isoform X1 [Manduca sexta]	Manduca	14	159	13	0.37
768409364	transcription elongation factor B polypeptide 2 [Plutella xylostella]	Plutella	2	5	1	0.37
768416674	D-erythrulose reductase-like [Plutella xylostella]	Manduca and Plutella	1	4	1	0.37

1714837793	endoplasmic reticulum chaperone BiP isoform X2 [Manduca sexta]	Manduca	32	494	4	0.36
1714788098	myosin regulatory light chain sqh [Manduca sexta]	Manduca	7	30	7	0.36
49532918	cellular retinoic acid binding protein [Plutella xylostella]	Plutella	5	86	4	0.36
768430181	putative RNA-binding protein 15 [Plutella xyl		4	14	4	0.36
1714829894	glycine cleavage system H protein, mitochondrial like [Manduca sexta]	Manduca	1	6	1	0.36
768445580	prefoldin subunit 2 [Plutella xylostella]	Plutella	1	8	1	0.36
768420307	prefoldin subunit 1 [Plutella xylostella]	Plutella	1	6	1	0.35

**Accession**	**Protein**	**Organism**	**# Peptides**	**# PSMs**	**# Unique Peptides**	**Fold change difference**
1714848962	charged multivesicular body protein 4b-like [Manduca sexta]	Manduca	3	29	3	0.35
1714827818	myosin heavy chain, muscle isoform X14 [Manduca sexta]	#N/A	169	2916	0	0.35
1714825657	stromal cell-derived factor 2 [Manduca sexta]	Manduca	1	6	1	0.34
768409789	CD2-associated protein isoform X1 [Plutella xylostella]	Plutella	1	1	1	0.34
768443011	ubiquitin-fold modifier-conjugating enzyme 1 [Plutella xylostella]	Plutella	3	14	3	0.34
1714801645	uncharacterized protein LOC115441686 [Manduca sexta]	Manduca	1	3	1	0.34
1714800627	dynein beta chain, ciliary [Manduca sexta]	Manduca	1	2	1	0.34
1025716978	thioredoxin-like protein [Plutella xylostella]	Plutella	2	100	2	0.34
768445306	aldehyde dehydrogenase X, mitochondrial-like [Plutella xylostella]	Plutella	9	77	3	0.33
768441902	U1 small nuclear ribonucleoprotein 70 kDa [Plutella xylostella]	Plutella	2	4	2	0.33
1714799717	transcriptional activator protein Pur-alpha isoform X2 [Manduca sexta]	Manduca	3	17	1	0.32
1714792353	uncharacterized protein LOC115456444	Manduca	2	5	2	0.32

	[Manduca sexta]					
1714785743	methanethiol oxidase [Manduca sexta]	Manduca	1	6	1	0.32
768445694	uncharacterized protein LOC105394551 [Plutella xylostella]	Plutella	2	8	2	0.32
1714823349	uncharacterized protein LOC115446507 [Manduca sexta]	Manduca	1	15	1	0.32
1714790157	protein lethal(2)essential for life-like [Manduca sexta]	Manduca	6	93	4	0.31
1714839079	ferritin subunit [Manduca sexta]	Manduca	1	9	1	0.31
1714789564	U6 snRNA-associated Sm-like protein LSm6 [Manduca sexta]	Manduca	2	9	2	0.31
1714782530	transcription elongation factor SPT5 [Manduca sexta]	Manduca	1	2	1	0.31
768418109	LDLR chaperone boca [Plutella xylostella]	Plutella	3	19	3	0.30
1714847321	myosin-2 essential light chain isoform X1 [Manduca sexta]	Manduca	5	31	5	0.30
768419118	glutenin, high molecular weight subunit 12-like [Plutella xylostella]	Plutella	3	37	3	0.30
1714844466	oxygen-dependent coproporphyrinogen-III oxidase isoform X1 [Manduca sexta]	Manduca	3	15	2	0.29
768432241	proteasome subunit beta type-2-like [Plutella xylostella]	Plutella	2	8	1	0.29
768410802	LOW QUALITY PROTEIN: calumenin-A-like [Plutella xylostella]	Plutella	3	11	2	0.29
1714822573	U6 snRNA-associated Sm-like protein LSm5 [Manduca sexta]	Manduca	1	3	1	0.29
768408389	probable pterin-4-alpha-carbinolamine dehydratase isoform X1 [Plutella xylostella]	Plutella	3	17	1	0.28
1714858987	60S ribosomal protein L35a [Manduca sexta]	Manduca	1	5	1	0.28
1714840451	multifunctional protein ADE2 [Manduca sexta]	Manduca	9	73	1	0.28
768427793	phosphatidylethanolamine-binding protein homolog F40A3.3-like [Plutella xylostella]	Plutella	4	38	4	0.28
768411157	classical arabinogalactan protein 4-like [Plutella xylostella]	Plutella	1	8	1	0.28

Accession	Protein	Organism	# Peptides	# PSMs	# Unique Peptides	Fold change differenc e
1714804609	charged multivesicular body protein 2a [Manduca sexta]	Manduca	2	6	2	0.28
1714822245	uncharacterized protein LOC115446254 [Manduca sexta]	Manduca	3	13	3	0.28
768420965	peptidyl-prolyl cis-trans isomerase B [Plutella xylostella]	Plutella	4	33	4	0.28
768436449	uncharacterized protein LOC105390160 [Plutella xylostella]	Plutella	4	16	4	0.27
768431892	EF-hand domain-containing protein D2 homolog [Plutella xylostella]	Manduca and Plutella	2	6	2	0.27
1714844694	alpha-centractin [Manduca sexta]	Manduca	5	13	5	0.27
768424642	chitooligosaccharidolytic beta-N acetylglucosaminidase [Plutella xylostella]	Plutella	1	2	1	0.26
768442159	WD repeat-containing protein 61 [Plutella xylostella]	Plutella	3	10	3	0.26
1714808268	sodium/calcium exchanger regulatory protein 1 [Manduca sexta]	Manduca	2	36	1	0.26
1714815435	cdc42 homolog [Manduca sexta]	Manduca	4	7	4	0.26
1714788570	FK506-binding protein 2 [Manduca sexta]	Manduca	9	59	1	0.26
768443236	pyridoxine-5'-phosphate oxidase-like [Plutella xylostella]	Plutella	1	5	1	0.26
768412859	LOW QUALITY PROTEIN: titin [Plutella xylostella]	Plutella	2	2	2	0.26
1714838887	zonadhesin-like isoform X1 [Manduca sexta]	Manduca	1	7	1	0.25
1714850655	pyruvate carboxylase, mitochondrial-like [Manduca sexta]	Manduca	7	43	1	0.25
1714840166	dynactin subunit 1 [Manduca sexta]	Manduca	1	2	1	0.25
768451365	LOW QUALITY PROTEIN: alpha-2-macroglobulin receptor-associated protein-like [Plutella xylostella Plutella		1	4	1	0.25
1714812934	U6 snRNA-associated Sm-like protein LSm8 [Manduca sexta]	Manduca	2	4	2	0.24
768444256	barrier-to-autointegration factor B [Plutella xylostella]	Plutella	3	15	3	0.24

1714793978	ubiquitin-conjugating enzyme E2 variant 2 [Manduca sexta]	Manduca	4	38	1	0.24
1714802054	glutathione S-transferase 2-like isoform X1 [Manduca sexta]	Manduca	1	6	1	0.24
1714800843	nuclear distribution protein nudE-like 1 isoform X1 [Manduca sexta]	Manduca	1	2	1	0.23
768412956	esterase E4-like isoform X1 [Plutella xylostella]	Plutella	1	7	1	0.23
1714806639	Y-box factor homolog [Manduca sexta]	Manduca	2	14	1	0.23
1714822508	protein yellow-like [Manduca sexta]	Manduca	2	4	2	0.22
1714853454	beta-1,3-glucan-binding protein 2-like [Manduca sexta]	Manduca	2	6	2	0.22
1714822611	ATP-dependent Clp protease ATP-binding subunit c[illegible] mitochondrial isoform X2 [Manduca sexta] Manduc[illegible]		3	12	3	0.21
1714810293	heat shock factor-binding protein 1 isoform X1 [Manduca sexta]	Manduca	6	14	6	0.21
1714810464	uncharacterized protein LOC115443743 isoform X1 [Manduca sexta]	Manduca	4	35	1	0.20
768418023	ATP-dependent RNA helicase dbp4-like [Plutella xylostella]	Manduca and Plutella	1	8	1	0.20

Accession	Protein	Organism	# Peptides	# PSMs	# Unique Peptides	Fold change difference
1714819501	ribosome production factor 2 homolog [Manduca sexta]	Manduca	1	2	1	0.20
768415969	carbonic anhydrase 15-like [Plutella xylostella]	Plutella	1	3	1	0.19
1714808375	alpha-tocopherol transfer protein-like [Manduca sexta]	Manduca	2	2	2	0.19
1714846832	selenoprotein M-like [Manduca sexta]	Manduca	1	4	1	0.19
1714792501	protein seele [Manduca sexta]	Manduca	4	15	4	0.18
768435469	coactosin-like protein isoform X1 [Plutella xylostella]	Manduca and Plutella	2	12	2	0.18
1714838135	sorting nexin lst-4 [Manduca sexta]	Manduca	2	7	2	0.18
1714840381	proteasome subunit beta type-1 [Manduca	Manduca	3	14	3	0.18

	sexta]					
1714830106	digestive cysteine proteinase 1 [Manduca sexta]	Manduca	3	14	1	0.18
768420530	tropomyosin-2 isoform X1 [Plutella xylostella]	#N/A	28	546	0	0.17
822092813	cytochrome b-c1 complex subunit Rieske, mitochondrial [Plutella xylostella]	Plutella	1	7	1	0.16
768408352	putative peptidyl-prolyl cis-trans isomerase dodo isoform X1 [Plutella xylostella]	Plutella	1	7	1	0.16
1714820997	thymosin beta isoform X1 [Manduca sexta]	Manduca	4	20	4	0.16
1714821490	alpha-N-acetylgalactosaminidase isoform X1 [Manduca sexta]	Manduca	2	4	2	0.14
1714835304	protein lethal(2)essential for life-like [Manduca sexta]	Manduca	3	27	1	0.14
768431908	myotrophin-like [Plutella xylostella]	Plutella	3	10	3	0.10
768445304	aldehyde dehydrogenase X, mitochondrial-like [Plutella xylostella]	Plutella	6	68	1	0.07
822092476	glyceraldehyde-3-phosphate dehydrogenase [Plutella xylostella]	Plutella	7	259	1	0.06
1714782947	uncharacterized protein LOC115444227 [Manduca sexta]	Manduca	8	20	5	0.05
1714789068	uncharacterized protein LOC115455834 [Manduca sexta]	Manduca	5	31	4	0.03
1714853738	uncharacterized protein LOC115453726 isoform X1 [Manduca sexta]	Manduca	4	17	4	0.03
822092754	uncharacterized protein LOC105388666 [Plutella xylostella]	Plutella	18	131	6	0.03
1174098354	basic juvenile hormone-suppressible protein 2-like precursor [Plutella xylostella]	Plutella	5	19	4	0.02
1714835288	heat shock protein 68-like [Manduca sexta]	Manduca	18	129	2	0.02
607029573	small heat shock protein [Plutella xylostella]	Plutella	3	24	2	0.02
1714790048	protein lethal(2)essential for life-like [Manduca sexta]	Manduca	3	9	3	0.02
1714785079	protein lethal(2)essential for life-like [Manduca sexta]	Manduca	5	35	3	0.01
1714794739	heat shock protein 68-like [Manduca sexta]	Manduca	5	42	2	0.01

Supplementary figure 1: Gene ontology enrichment analysis of biological process (upregulated and downregulated) of *T. absoluta* proteins after entomopathogenic fungal infection. Figure shows up regulated biological process (i) and down regulated biological process (ii) after *B. bassiana*-FC21 treatment; up regulated biological process (iii) and down regulated biological process (iv) after *P. lilacinum*-FC18 treatment. In figure A. Dot plot represents the up and regulated biological process in *T. absoluta;*.B. Heatmap represents the proteins involved in the up and down regulated biological process; C. Enrichment map represents the enriched biological process of up and down regulated proteins; D. Upset plot represents the up and down regulated biological process and the number of proteins common among the biological process.

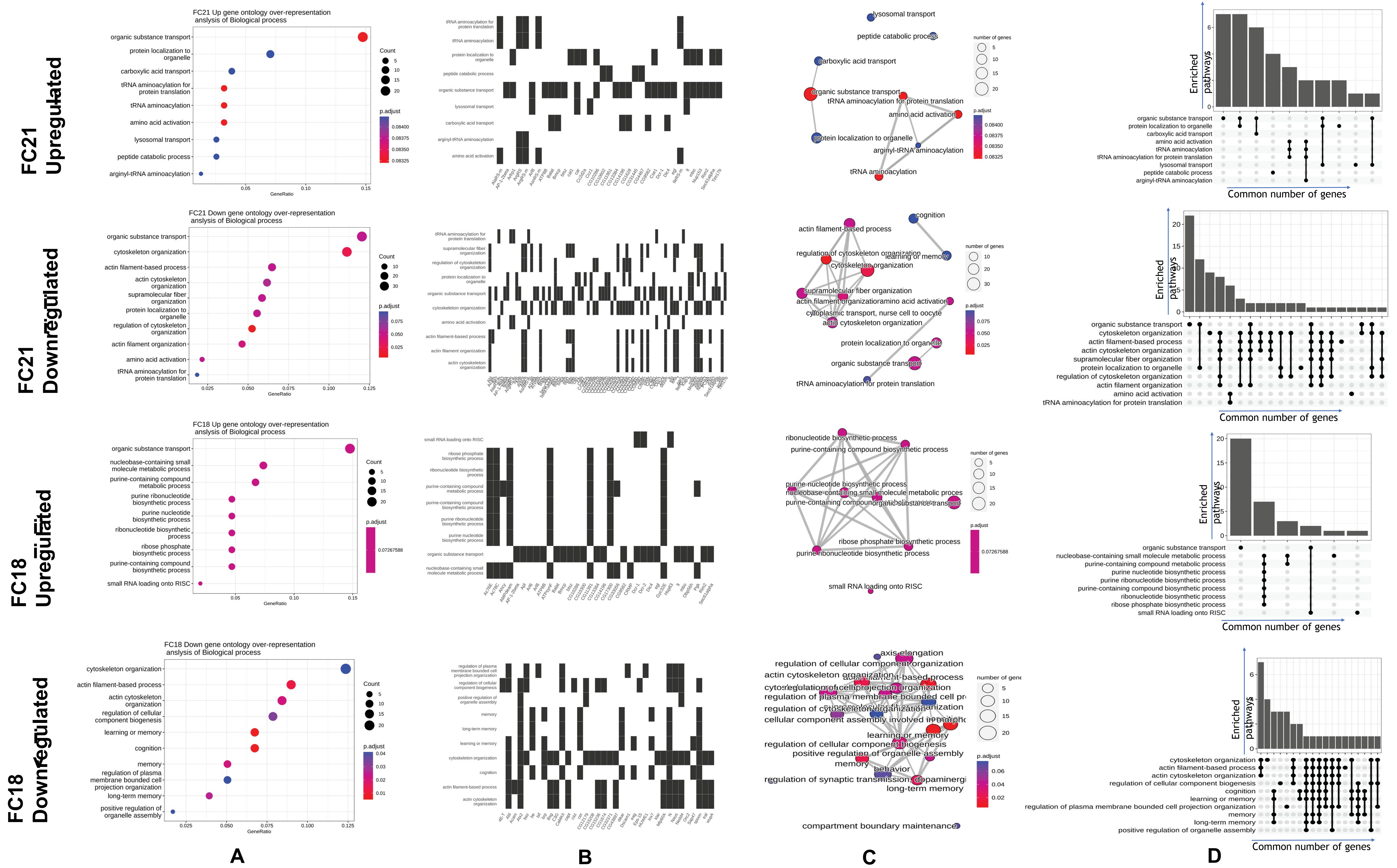
FC21 Upregulated
FC21 Downregulated
FC18 Upregulated
FC18 Downregulated
FC21 Up gene ontology over-representation analysis of Biological process
organic substance transport
protein localization to organelle
carboxylic acid transport
tRNA aminoacylation for protein translation
tRNA aminoacylation
amino acid activation
lysosomal transport
peptide catabolic process
arginyl-tRNA aminoacylation
GeneRatio
Count
p.adjust
FC21 Down gene ontology over-representation analysis of Biological process
cytoskeleton organization
actin filament-based process
actin cytoskeleton organization
supramolecular fiber organization
regulation of cytoskeleton organization
actin filament organization
FC18 Up gene ontology over-representation analysis of Biological process
nucleobase-containing small molecule metabolic process
purine-containing compound metabolic process
purine ribonucleotide biosynthetic process
purine nucleotide biosynthetic process
ribonucleotide biosynthetic process
ribose phosphate biosynthetic process
purine-containing compound biosynthetic process
small RNA loading onto RISC
FC18 Down gene ontology over-representation analysis of Biological process
regulation of cellular component biogenesis
learning or memory
cognition
memory
regulation of plasma membrane bounded cell projection organization
long-term memory
positive regulation of organelle assembly
number of genes
cytoplasmic transport, nurse cell to oocyte
axis elongation
regulation of cellular component organization
behavior
regulation of synaptic transmission, dopaminergic
compartment boundary maintenance
Enriched pathways
Common number of genes
A
B
C
D

Supplementary figure 2: KEGG pathway analysis proteins involved in amino acid tyrosine metabolism.

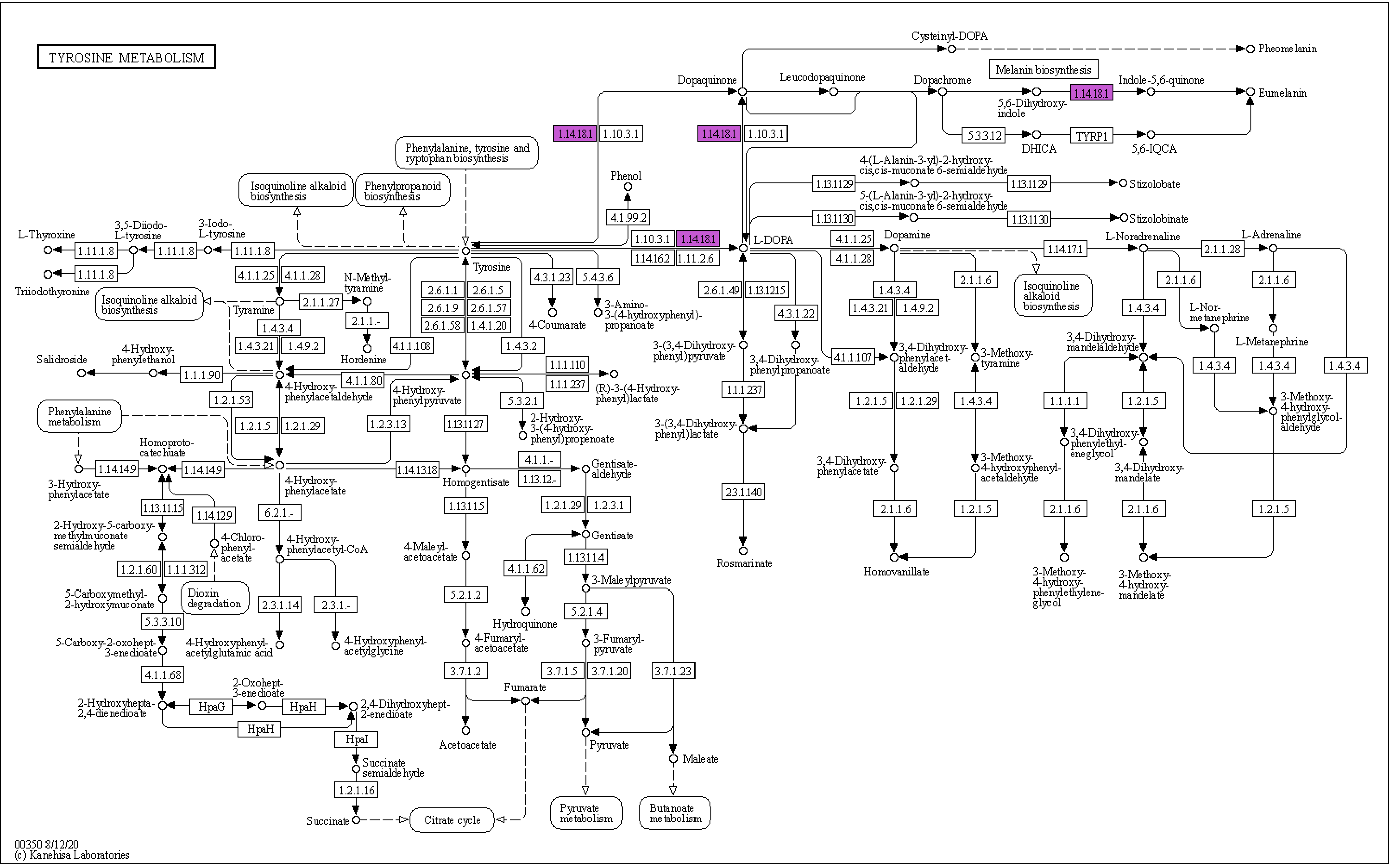

Supplementary table: 1: Proteins identified which were involved in immune system of *T. absoluta* after *B.bassiana* (FC21) infection and their expression profile after 48 h of post- infection

**Protein name and molecular weight of the protein (kDa)**	**2-fold change difference of protein expression after 48 h of infection**
Phenoloxidase activating factor 2-like (50.5)	(0.42)
beta-1,3-glucan binding protein (53.6)	(0.49)
beta-1,3-glucan binding protein 2-like (54.4)	(0.22)
P450 CYP6 Family protein 6 (60.2)	(3.07)
Cytochrome P450 9e2-like (61.3)	(2.57)
Glutathione-S-transferase 2 isoform like (26.5)	(0.24)
Esterase E4-like isoform X1(63.3)	(0.23)
Thioredoxin like protein (11.7)	(0.34)
Peroxiredoxin (27.7)	(0.49)
Catalase (56.8)	(0.48)
Catalase like (64.6)	(0.46)
Ferritin Subunit (24)	(0.31)

Supplementary table: 2: Proteins identified which were involved in immune system of *T. absoluta* after *P. lilacinum* (FC18) infection and their expression profile after 48 h of post-infection.

**Protein name and molecular weight of the protein (kDa)**	**2-fold change difference of protein expression after 48 h of infection**
Phenoloxidase activating factor 2-like (50.5 kDa)	(0.36)
Prophenoloxidase 1 (78.4 kDa)	(0.24)
Beta-1,3-glucan binding protein (53.6 kDa)	(0.30)
P450 CYP6 Family protein 10 (58.8 kDa)	(2.11)
P450 CYP6 Family protein 6 (60.2 kDa)	(2.23)
Cytochrome P450 9e2-like (61.3 kDa)	(2.00)
Glutathione-S-transferase 2 like isoform (26.5 kDa)	(0.35)
Thioredoxin like protein (11.7 kDa)	(0.33)
Thioredoxin like protein 1 (31.6 kDa)	(0.44)
Peroxiredoxin 6 (25.1 kDa)	(0.40)
Transferrin (75.2 kDa)	(0.31)
Ferritin Subunit (24 kDa)	(0.39)
Lysozyme-like (16.4 kDa)	(0.14)
